# Imogolite nanotube modifications impact pulmonary toxicity in mice: implications for safe and sustainable by design (SSbD)

**DOI:** 10.1186/s12951-025-03647-w

**Published:** 2025-08-18

**Authors:** Pernille Høgh Danielsen, Sarah Søs Poulsen, Alicja Mortensen, Trine Berthing, Dorra Gargouri, Arianna Filoramo, Pekka Kohonen, Roland Grafström, Fabienne Testard, Ulla Vogel

**Affiliations:** 1 National Research Centre for the Working Environment (NFA), 105 Lersø Parkallé, Copenhagen Ø, Denmark; 2 Université Paris-Saclay, CEA, CNRS, NIMBE, LIONS, Gif-sur- Yvette, 91191 France; 3 Institute of Environmental Medicine, Karolinska Institutet, Nobels väg 13, Stockholm, 17177 Sweden; 4 Department of Toxicology, Misvik Biology, Karjakatu 35 B, Turku, 20520 Finland; 5 National Food Institute, Technical University of Denmark, Anker Engelunds Vej 1, Kgs, Lyngby, Denmark

**Keywords:** Inflammation, Imogolites, HARN, Acute phase response, Safe-by-design, Pulmonary exposure

## Abstract

**Background:**

Imogolite is a naturally occurring hollow aluminosilicate nanotube with potential for engineered applications due to its high aspect ratio, hydrophilicity, and polarization. However, these same features raise concerns about potential adverse health effects. These concerns parallel those associated with multi-walled carbon nanotubes (MWCNTs), which are known to cause inflammation, fibrosis, and cardiovascular effects. The purpose of this study was to investigate how surface functionalization of imogolite influences its toxicity and biological response, with the aim of informing safer design of nanomaterials. Female C57BL/6J mice were exposed via intratracheal instillation to 6, 18, or 54 µg of hydroxylated (Imo-OH) or methylated (Imo-CH_3_) imogolite. Toxicity was assessed at day 1, 28 and 90 post-exposure, with carbon black (Printex90) nanoparticles as a benchmark. Pulmonary inflammation and systemic acute-phase response were assessed as key indicators of chronic health effects.

**Results:**

Physicochemical characterization showed that Imo-OH dispersed as single nanotubes, while Imo-CH_3_ formed bundles, impacting surface accessibility. Both variants induced strong pulmonary inflammation, but Imo-OH elicited a stronger and more persistent neutrophil influx, lymphocyte recruitment, and acute-phase response. Cytotoxicity was low, though elevated total protein in bronchoalveolar lavage fluid indicated altered alveolar-capillary barrier integrity, especially for Imo-OH. Lung histopathology confirmed more severe lung lesions, macrophage aggregates, and type II pneumocyte hyperplasia in the Imo-OH group. Benchmark dose modeling revealed that Imo-OH’s inflammatory potential surpassed other high aspect ratio nanomaterials.

**Conclusions:**

Both imogolite variants induced pulmonary inflammation and an acute-phase response in mice; however, these effects were markedly reduced for the methylated imogolite (Imo-CH_3_). In addition to surface functionalization, factors like bundle formation and by-product particles may also influence toxicity. These findings emphasize the pivotal role of surface chemistry—and associated structural properties—in shaping the biological response to nanomaterials, reinforcing the need for thoughtful design strategies to promote safer applications in nanotechnology.

**Graphical abstract:**

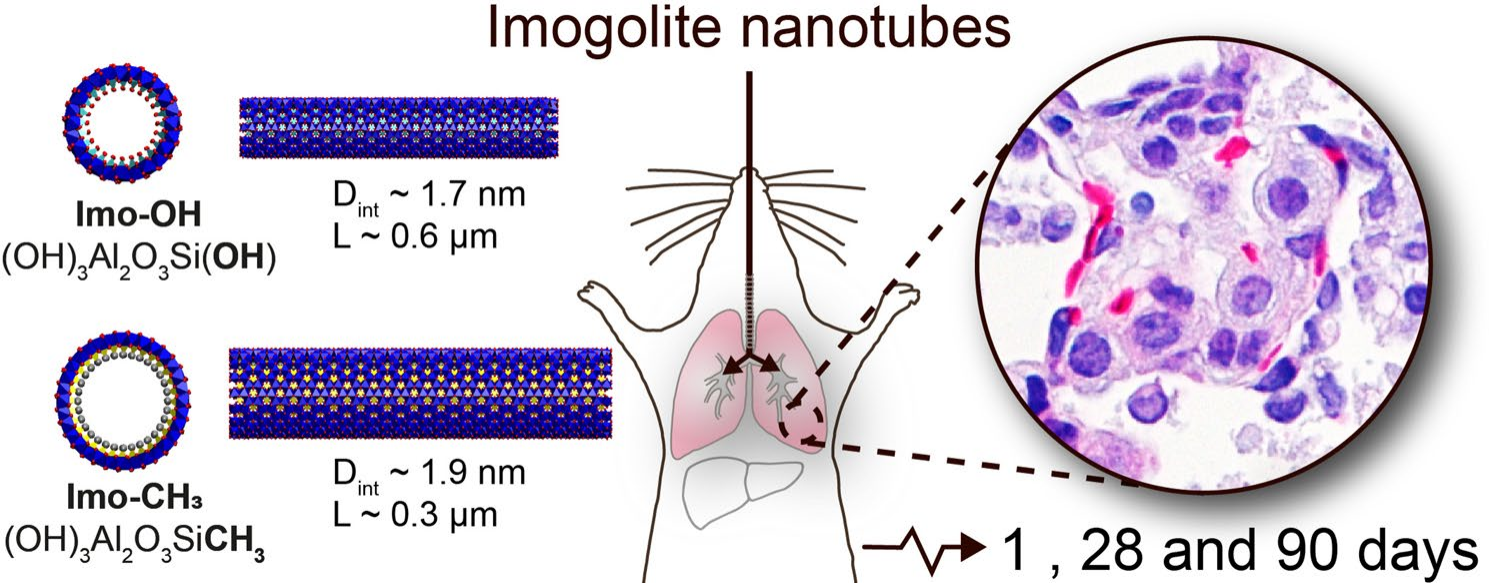

**Supplementary Information:**

The online version contains supplementary material available at 10.1186/s12951-025-03647-w.

## Background

Imogolite is a naturally occurring hollow, nanoscale aluminosilicate mineral primarily found in volcanic ash soils, but can also be chemically synthesized, enabling the production of both pristine and modified derivatives [1, 2]. Due to its distinctive structural and chemical properties, imogolite finds application in various fields, including catalysis, insulation, gas adsorption, membrane filtration, polymer-based nanocomposites, and flame retardants [3–5]. These applications are largely attributed to its nanotubular morphology, characterized by a high aspect ratio and properties like nanocavities, hydrophilicity, polarization, and a large band gap [6, 7].

However, the high aspect ratio of imogolite nanotubes raises health concerns particularly regarding inhalation exposure in occupational settings. Research on similar high aspect ratio nanotubes, especially multi-walled carbon nanotubes (MWCNTs), has shown that inhalation in rats can lead to chronic inflammation, fibrosis, and lung cancer [8, 9]. In mice, pulmonary exposure to MWCNTs has shown to cause inflammation, fibrosis, genotoxicity, plaque progression, and acute-phase responses [10–17], which are associated with increased risk of cardiovascular disease [18, 19].

To enhance the functionality of imogolite for specific applications, its intrinsic properties are often modified. For instance, the naturally hydroxylated inner surface can be functionalized with methyl to produce hybrid forms [5, 20]. Substituting silica with heavier atoms like germanium (Ge) can also alter material properties, as demonstrated in Ge-substituted imogolites [21]. While such modifications could enhance functionality, they may also affect toxicity, underscoring the need for comprehensive assessments.

Despite its potential, the toxicological data on imogolites remain limited. Ishikawa et al. investigated cellular responses to imogolite assemblies in human (Saos-2) and mouse (MC3T3-E1) osteoblast-like cells [22, 23]. Cells were seeded and cultured on scaffolds of imogolite and the findings indicated biocompatibility with enhanced proliferation and osteoblastic differentiation. Liu et al. assessed the cytotoxicity and genotoxicity of Ge-substituted imogolites in human fibroblast cell cultures, focusing on nanotube length [24]. While uptake was higher for shorter imogolites, no cytotoxicity was observed regardless of the length. However, genotoxicity - DNA damage driven by oxidative stress - where more pronounced with shorter imogolites. Rotoli et al. studied macrophages and airway epithelial cells exposed to imogolites, MWCNTs, and single-walled carbon nanotubes (SWCNTs) [25]. Imogolites were internalized by macrophages, but low cytotoxic and genotoxic effect with no clear dose-response relationship was shown. This low toxicity was attributed to their highly hydrophilic surface and the Al(OH)Al groups at the outer surface and SiOH groups at the inner surface [25]. Van den Brule et al. studied the toxicity of Ge-substituted imogolite in rats pulmonary exposed by intratracheal instillation [26]. They showed that Ge-based imogolites persisted in the lung and promoted lung inflammation, genotoxicity and fibrosis, challenging the conventional 5 μm threshold for fiber-induced pulmonary toxicity of high aspect ratio nanomaterials (HARN) [26]. Collectively, these studies demonstrate the need for standardized toxicity tests that consider particle length, chemical substitution, and surface modifications. Clearly, there is a need to alleviate potential health risk and therefore a Safe and Sustainable by Design (SSbD) strategy is essential. SSbD promotes early integration of safety considerations into material development, aiming to minimize toxicity at both molecular and macroscopic levels.

In this study, we investigate the pulmonary toxicity of two imogolite variants − hydroxylated (Imo-OH) and methylated (Imo-CH_3_) − in mice following intratracheal instillation. Specifically, we assess how increasing material complexity affects pulmonary inflammation and systemic acute-phase responses, which are key indicators of chronic health effects such as fibrosis, cancer, and cardiovascular disease. Our findings are discussed in the context of the physicochemical properties of these materials and their potential agricultural applications [27].

## Methods

### Imogolite nanotubes

The imogolites used in this study were synthesized at the PRODIGE facility of CEA, Paris-Saclay [28]. Imo-OH is by itself a multicomponent nanotube composed of various atoms (Si, Al, O, H), with a chemical structure that can be represented from the outer to the inner surface as (HO)_3_Al_2_O_3_Si(OH). Both surfaces are hydrophilic, with the external surface bearing Al_2_-µOH groups, while the inner surface is lined with Si-OH functionalities. The functionalization of the internal cavity with methyl moieties (Imo-CH_3_) increases the chemical complexity (Si, Al, O, H, C) [5]. This hybrid imogolite retains a similar outer surface but has a hydrophobic inner cavity fully covered by methyl groups, resulting in a slightly larger diameter than Imo-OH. The selective addition of carbon in the structure modifies the imogolite’s properties.

Both Imo-OH and Imo-CH_3_ were synthesized through the controlled hydrolysis of aluminum and silicon-based precursors, following protocols previously described [5, 29, 30]. For Imo-OH, tetraethoxysilane and aluminum chloride were used as precursors, and synthesis was performed at semi-pilot scale in 150 L of water. For Imo-CH_3_, trimethoxymethylsilane and aluminum-sec-butoxide were used as precursors, and synthesis was performed at lab scale in a 1 L Teflon reactor. The protocol is provided in the Supplementary Material.The characterization parameters are shown in Table 1.

**Table 1:** Characterization parameters

Sample				Mean length (Lm, nm) from AFM^#^ or TEM^$^ Lognormal distribution (Ln, σ)			Si/Al From XPS^+^
	Inner diameter (nm)	Outer diameter (nm)	BET (m^2^/g)	Lm (nm)	Ln (nm)	σ	
Imo-OH	1.70*	2.90*	407	653^#^	590.0	0.45	0.5
Imo-CH_3_	1.88	3.08	625	309^$^	301.8	0.21	0.55

*The dimension of the diameter measured by SAXS are slightly higher than classical values obtained for Imo-OH where internal and external diameter are closer 1.5 and 2.7 nm, respectively (Liao et al. 2018, Picot et al. 2024). These diameters being determined by SAXS, it is an indication of the presence of proto-imogolites and allophane in equilibrium with the Imo-OH

### Instillation suspensions

The instillation suspensions were prepared, following a standard protocol for toxicity studies of nanomaterials [31], at a concentration of 3.24 mg/ml in Nanopure water with 2% mouse serum and probe-sonicated on ice for 16 min at 10% amplitude without pause, using a Branson Sonifier S-450D (Branson Ultrasonics Corp., Danbury, CT, USA) equipped with a disruptor horn (model number 101-147-037). The suspensions were prepared form a stock Imo-OH suspension in milliQ water at 8.46 mg/ml or directly from the dried powder for Imo-CH_3_. The stock suspensions were then diluted to concentrations of 1.08, 0.36 and 0.12 mg/ml, corresponding to 54, 18 and 6 µg per 50 µl, respectively, and further sonicated for 4 min. Printex90, at a dose of 162 µg per mouse, was included as a reference material with known pulmonary toxicity. The vehicle, consisting of Nanopure water with 2% mouse serum, was similarly sonicated. The instillation volume per mouse was 50 µl.

### Animals and exposure

Seven weeks old female C57BL/6JRj mice (Janvier Labs, France), were randomly divided into four experimental groups. The housing conditions followed those described by Jacobsen et al. [32]. In brief, mice were housed under the controlled environmental conditions with a 12-hour light/12-hour dark cycle, a room temperature of 22 °C ± 1 °C, and a relative humidity of 55% ± 5%. They had free access to feed (Altromin1324, Brogaarden, Denmark) and water. The mice were housed in groups of three or six per polypropylene cage with Enviro-dri bedding (Brogaarden, Denmark) along with wood blocks and hides as enrichment items. At the start of experiment, the mice were anaesthetized and administered Imo-OH, Imo-CH_3_, Printex90 carbon nanoparticles, or vehicle (2% mouse serum (v/v) in water) through a single intratracheal instillation [33–36].

The experiment was conducted in overlapping series with terminations at day 1, 28 and 90 post-exposure. Each series included vehicle control groups and groups of mice exposed to the particle materials. The sample size for each dose group at each time point was *n* = 6 for markers of lung toxicity. In addition, separate groups of *n* = 5 animals were allocated for histology, with a single dose group and termination at 28 and 90 days post exposure.

The experimental series also included particle materials not covered in this study. As a result, the cumulative number of vehicle control animals was *N* = 40 at day 1, and *N* = 27 at both 28 and 90 days post-exposure. The cumulative number of animals exposed to Printex90 was *N* = 12 at day 1, and *N* = 18 at both 28 and 90 days post-exposure.

The study was approved by the Danish Animal Experiment Inspectorate (permission no. 2020-15-0201-00485) and received prior approval from the local Animal Ethics Council.

### Collection of plasma, tissue and BAL cells

Mice were euthanized at 1 day, 28 days, or 3 months post-exposure via intramuscular injection of 0.1 ml ZRF-solution (Zoletil 250 mg, Rompun 20 mg/ml, Fentanyl 50 mg/ml in sterile isotonic saline). Heart blood was withdrawn via intracardiac puncture, stabilized with K_2_EDTA and centrifuged to collect the plasma, which were stored at − 80 °C. Small pieces of lung and liver tissues were snap-frozen in liquid nitrogen and stored in cryotubes at − 80 °C until RNA isolation for mRNA expression analysis. Bronchoalveolar lavage fluid (BALF) was collected by flushing the lungs twice with 1 ml saline through the trachea. The BALF was kept on ice until separation of fluid and cells by centrifugation at 400 x *g* for 10 min at 4 °C. The separated BAL cells were re-suspended in HAMF12 medium with 10% fetal bovine serum. The total number of BAL cells and cell viability were measured using the NucleoCounter NC-200 (Chemometec, Allerød, Denmark). BAL differential cell counts of macrophages, neutrophils, lymphocytes, and eosinophils were assessed by counting 200 cells per sample under a light microscope [31].

### Total protein and LDH in BAL fluid

The Pierce™ BCA Protein Assay Kit (Thermo Fisher Scientific Inc.) was used to measure total protein content in BALF [14]. The Lactate Dehydrogenase Assay Kit (Sigma-Aldrich MAK464 from Merck KGaA, Darmstadt, Germany) was used to measure LDH activity in BALF.

### mRNA expression of *Saa3* in lung tissue and *Saa1* in liver tissue

Gene expression levels were measured by quantitative real-time reverse transcriptase polymerase chain reaction (RT-PCR) as previously described [15, 37, 38]. The acute phase response was assessed by measuring *Saa3* and *Saa1* mRNA expression levels in lung and liver tissues, respectively. Total RNA was isolated using the Maxwell^®^ 16 LEV simplyRNA Tissue Kit (AS1280, Promega, USA) according to the manufacturer’s protocol. Complementary DNA (cDNA) was prepared using TaqMan^®^ reverse transcription reagents (Applied Biosystems, USA) following the manufacturer’s instructions. Total RNA and cDNA concentrations were measured with the NanoDrop 2000c (ThermoFisher, USA). Gene expression levels were determined using predesigned TaqMan expression assays with 18 S rRNA as the endogenous control (Applied Biosystems, USA). Samples were run in triplicates on the ViiA7 Real-Time PCR detector (Applied Biosystems, USA). Negative controls were included in each analysis run. The relative expression of the target genes was measured using the comparative CT method.

### SAA3 protein levels in plasma

The Mouse SAA3 ELISA kit (Millipore EZMSAA3 from Merck KGaA, Darmstadt, Germany) was used to measure SAA3 protein levels in plasma following the manufacturer’s protocol [15]. At the day 1 post-exposure time point, all doses were analyzed, while only the highest dose (54 µg/mouse) was analyzed at the day 28 post-exposure time point. Control groups (*n* = 6) were randomly selected to represent the different exposure days.

### Histological examination of lung and liver tissue

At necropsy on days 28 and 90 post-exposure, lungs and liver samples were collected from six vehicle control mice and five mice from Imo-OH and Imo-CH_3_ high-dose groups (54 µg/animal). For lung fixation, the trachea was cannulated, and 4% neutral buffered formaldehyde was infused at a constant pressure of 25 cm before opening the thorax. The lungs were then excised and immersed in the same fixative. Liver samples were collected after inspecting the abdominal cavity and fixed by immersion in 4% neutral buffered formaldehyde. Tissue sampling and trimming followed established guidelines for organ processing in rodents [39, 40]. After fixation, lung and liver samples were embedded in paraffin, sectioned at 2–3 μm thickness, and stained with hematoxylin and eosin (HE) for histological examination. Light microscopy analysis was conducted on all control and high-dose groups by a single operator, first with knowledge of treatment groups, followed by a blinded evaluation [41]. The diagnostic nomenclature for microscopic changes adhered to INHAND recommendations for the lungs [42] and liver [43].

For focal mononuclear cell infiltrations, laden macrophage aggregates, and macrophage-dense areas, both the number and severity of changes were reported separately for the left and the right lung (cranial, middle accessory, and caudal lobes). Mononuclear cell infiltrations were defined as regions with an increased density of mononuclear cells compared to the surrounding tissue, forming distinct shapes and sizes. Only infiltrates containing more than 20 cells were counted. These infiltrations primarily consisted of lymphocytes but occasionally included other inflammatory cells and were observed near blood vessels, bronchioles, alveolar ducts, interstitial regions or subpleural areas. Laden macrophage aggregates were defined as clusters of alveolar macrophages in close proximity at a single site. In contrast, areas of laden macrophages were defined as lung regions with an increased density of alveolar macrophages compared to the background, but where macrophages were not clustered together.

In the liver, inflammatory cell infiltrates (focal mononuclear cell infiltrations) were categorized as small (≤ 10 inflammatory cells, occasionally accompanied by necrotic hepatocytes with distinct eosinophilic cytoplasm) or large (> 10 inflammatory cells, often surrounding necrotic hepatocytes with distinct eosinophilic cytoplasm, with or without apoptotic bodies or debris). Liver changes were assessed for severity, including microvesicular steatosis, an apparent increase in binucleate hepatocytes and Kupffer cells, hyperplasia of connective tissue near bile ductules, and oval cell hyperplasia.

The severity of histopathological changes in both the lungs and liver was graded on a five-point scale Grade 1: minimal/very few/very small; Grade 2: mild/few/small; Grade 3: moderate/moderate number/moderate size; Grade 4: marked/many/large; Grade 5: massive/extensive number/extensive size.

### Statistical analyses, exploratory data analyses

Statistical analyses were performed using the software package GraphPad Prism 8.0.2 (GraphPad Software Inc., La Jolla, CA, USA). Data were tested for normality using the Shapiro-Wilk test, for variance homogeneity using the Brown-Forsythe test, and each dose group was compared with the vehicle control group by ordinary one-way ANOVA with Dunnett’s multiple comparison test. Data that did not fulfill normality and variance homogeneity criteria were analyzed by the nonparametric Kruskal-Wallis test. Pairwise statistical comparisons between Imo‑OH and Imo‑CH_3_ for each dose group were performed using Sidak’s multiple comparisons test. Gene expression data were logarithmically transformed before analysis. P-values ≤ 0.05 were considered significant.

### Statistical analyses, benchmark dose analyses (BMD)

Benchmark dose analyses were performed with the ToxicR R package (v. 23.4.1.1.0). The neutrophil values were log2-transformed prior to the BMD analysis for each dose, including control. If the value was empty, it was replaced with the total number of cells/400, as that was the lowest limit of the detection range. The BMD analysis used the robust median and median absolute deviation (MAD) values as inputs, as well as n or the number of observations. BMDs were obtained as a weighted average of exponential 3, exponential 5, Hill’s and power models fitted with the Laplace method and a benchmark response (BMR) of one standard deviation. The mass dose levels, and the surface area dose levels were modelled separately. The BMD and the 95% lower and upper limits were obtained (BMDL and BMDU). BMD values 20-fold greater or lesser than the measured concentrations were set to these limits.

## Results

### Imogolite characterization

**Fig. 1:**
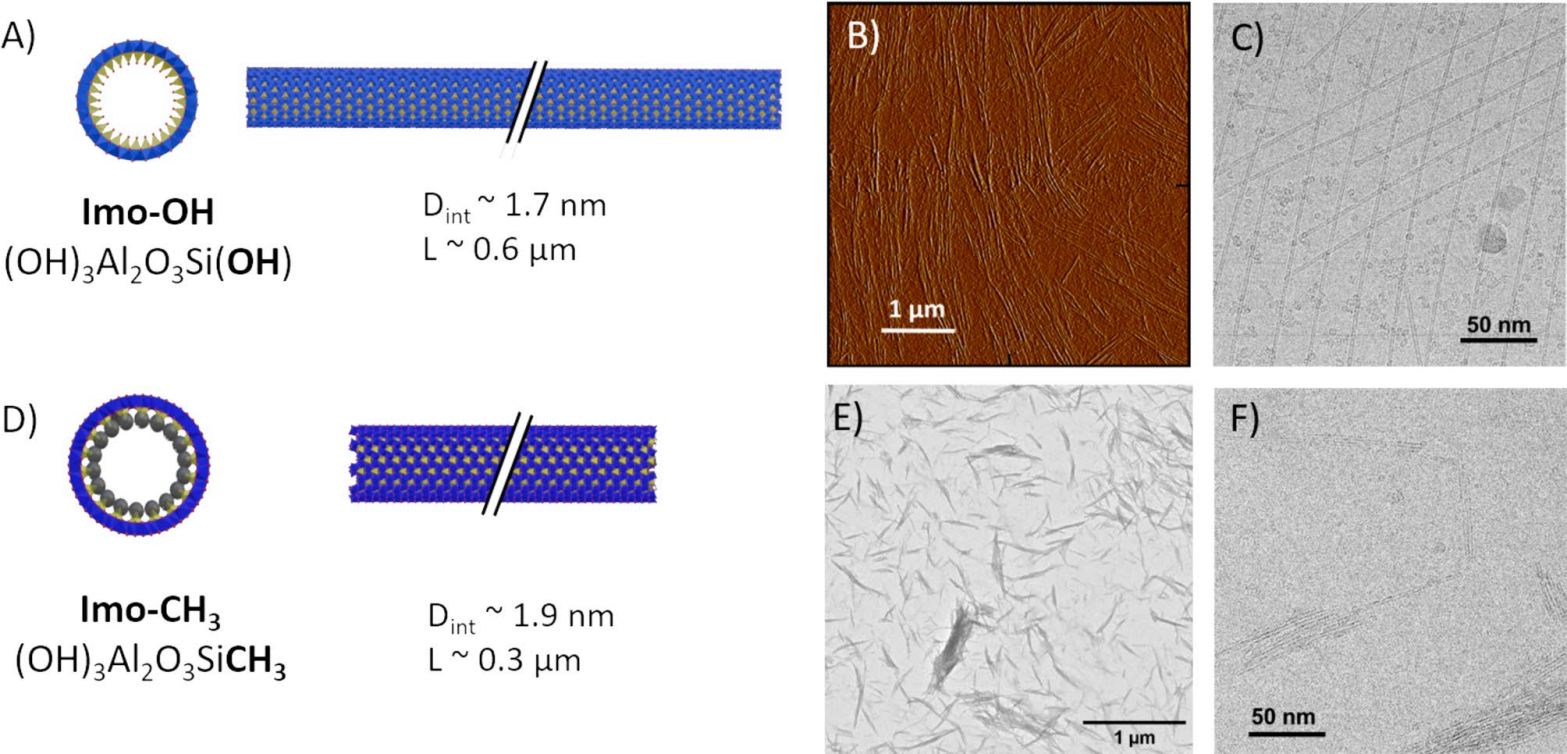
(**A**) Schematic representation of Imo-OH is shown with its chemical formula, internal diameter (D_int_), and average length (L), including both transversal and side view. Atoms are represented as spheres in red, light blue, and blue corresponding to oxygen, silicon, and aluminum, respectively. (**B**) Example of an AFM images and (**C**) a cryoTEM image of Imo-OH is shown. (**D**) Schematic representation of Imo-CH_3_ with its chemical formula, the internal diameter (D_int_) and the average length (L), including both transversal and side view with. Atoms are represented as spheres in red, yellow, blue and grey, corresponding to oxygen, silicon, aluminum and carbon, respectively. (**E**) Example of a TEM images and (**F**) a cryoTEM image of Imo-CH_3_ is shown

Imogolites were analyzed prior to their use using a combination of techniques (atomic force microscopy (AFM), transmission electron microscopy (TEM), small-angle X-ray scattering (SAXS), transmission electron cryomicroscopy (cryoTEM)) to determine chemical composition, physical dimensions, specific surface areas and, agglomeration state (Table 1; Fig. 1, Fig. S1-S6, Table S1 and S2). X-ray photoelectron spectroscopy (XPS) identified the characteristic Si/Al ratio of imogolites, interpretation of XPS for imogolites is rather difficult because of the wall polarization [7]. Infrared spectroscopy (IR) identified the characteristic doublet between 1000–900 cm^−1^ (Si-O-Al stretching) of the nanotube structure and the typical bands at 781 and 1275 cm^−1^ of Si-C and Si-CH_3_ respectively for the Imo-CH_3_ [30, 44] (See Fig. S1 and Fig. S2 in the supplementary material for the detailed IR and XPS spectra, respectively). XPS spectra (survey and core level) of Imo-OH and Imo-CH_3_, obtained from dried powder and the table of the peak attribution issued from fitting are shown in the supplementary material (Fig. S3, S4, S5, and Table S2). The deconvolution of Si 2p core level spectra and C 1 s spectra clearly identified the differences between both imogolite types and evidence the Si-C bound typical in Imo-CH_3_. SAXS patterns (Fig. S6) describe a dispersion of nanotubes which can be fitted using a homogeneous cylinder core-shell model including the association of 1 to 4 tubes in a bundle, to extract the internal and outer diameters [7, 45]. The parameters used for fitting the SAXS patterns are shown in Table S2. For Imo-OH, the obtained values are slightly higher than classical values reported in the literature, indicating the presence of allophanes and proto-imogolites [29, 46]. These smaller particles (nanospheres and nanotiles) with imogolite-like structure are in equilibrium with the imogolites. They are clearly visible in the cryoTEM images (Fig. 1C), although in many images they represent only a small fraction of the sample by mass. The SAXS patterns also indicate that Imo-CH_3_ tends to form bundles, while Imo-OH is more easily dispersed as single nanotubes (Fig. S2). This feature is confirmed in the cryoTEM images, where Imo-CH_3_ predominantly appears as bundles (Fig. 1F). The precise quantification of the bundles size distribution could be done following a systematic X-ray scattering and cryoTEM approach [47]. However, for this study, we limit the analysis to the accessible specific surface area to take into account the formation of bundles. The formation of bundles has a significant impact on the specific surface areas accessible to biological moieties. Specific surface areas are typically measured by BET analysis on dried powder; however, Dhaini et al. demonstrated that these measurements underestimate the true surface area due to the formation of large bundles, which prevents access to the nitrogen N_2_ gas molecules [48]. Additionally, they emphasized the different contributions of intratube, intertubes, and interbundles surfaces to the total surface area, as well as differences in their accessibilities. Therefore, the use of BET-measured surface areas should be interpreted with caution when comparing the effects of the imogolite suspensions.

### Inflammation

**Fig. 2:**
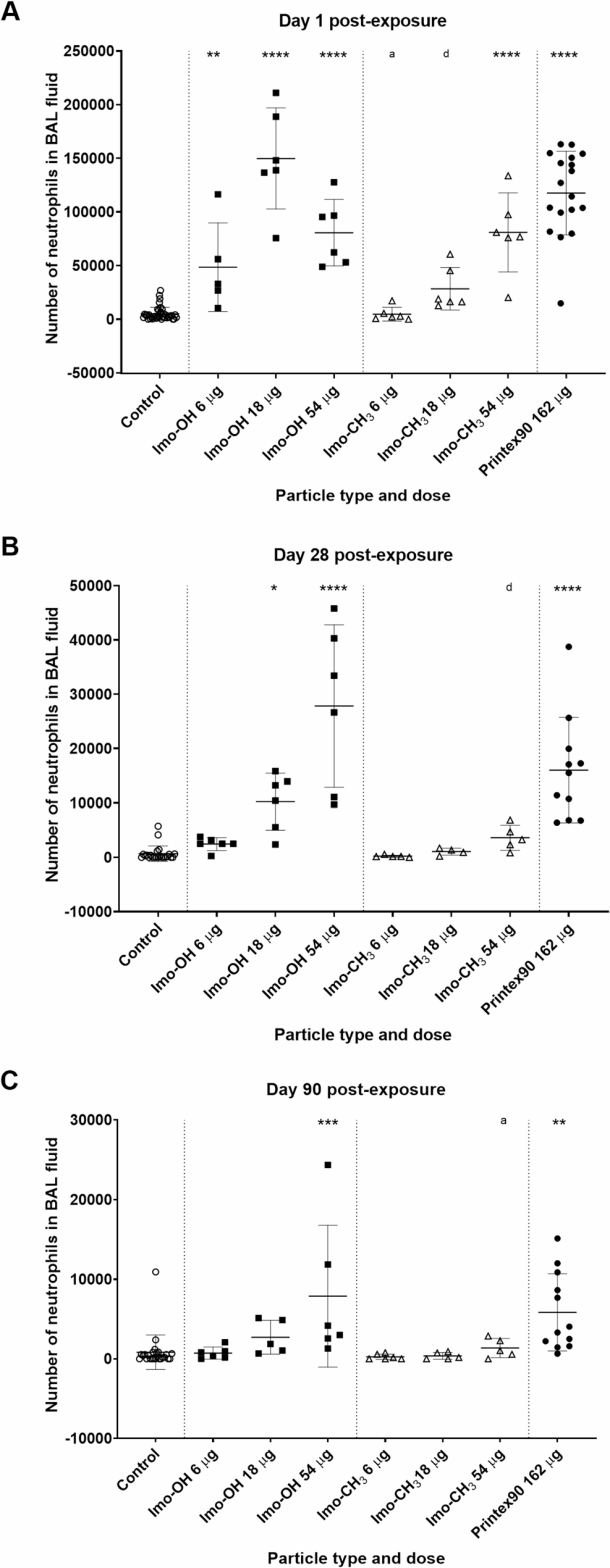
Neutrophil influx (**A**) 1, (**B**) 28 and (**C**) 90 days post-exposure to imogolites. Data are shown as individual values, mean ± SD. Stars indicate comparison with the control (Dunnett’s multiple comparison method), and letters indicate comparison to the same dose of Imo-OH (Sidak’s multiple comparison test). Significance levels are as follows: * or a, P ≤ 0.05; ** or b, P ≤ 0.01; *** or c, P ≤ 0.001; **** or d, P ≤ 0.0001

Both types of imogolite triggered a significant pulmonary inflammatory response, primarily characterized by an increase in the total cell number (Supplemental Table S3), largely driven by a dose-dependent increase in neutrophils on day 1 post-exposure (Fig. 2A). Imo-OH induced a strong inflammatory response across all dose levels, while Imo-CH_3_ showed dose-dependency with a similar significant effect at the highest dose (Fig. 2A). Imo-OH also exhibited a prolonged inflammatory response, with notably elevated neutrophil levels persisting through day 28 and 90 post-exposure (Fig. 2B and C and Supplementary Table S3). Additionally, Imo-OH caused a significant increase in lymphocytes and eosinophils on day 1 post-exposure (Supplemental Table S3), this effect was only observed at the middle dose, resulting in a non-monotonic (inverted) dose-response pattern. This pattern aligns with findings from previous studies on carbon nanotube exposure [49, 50]. Elevated lymphocyte levels were also observed on day 28 (middle dose) and day 90 (highest dose) post-exposure (Supplemental Table S3).

### Cytotoxicity and total protein levels in BAL fluid

Cytotoxicity remained largely unaffected, there were no significant change in cell viability at any post-exposure time points (Supplemental Fig. S7) and increased LDH activity was only significantly increased at the highest dose of Imo-OH at day 28 post-exposure (Supplemental Fig. S8B).

**Fig. 3:**
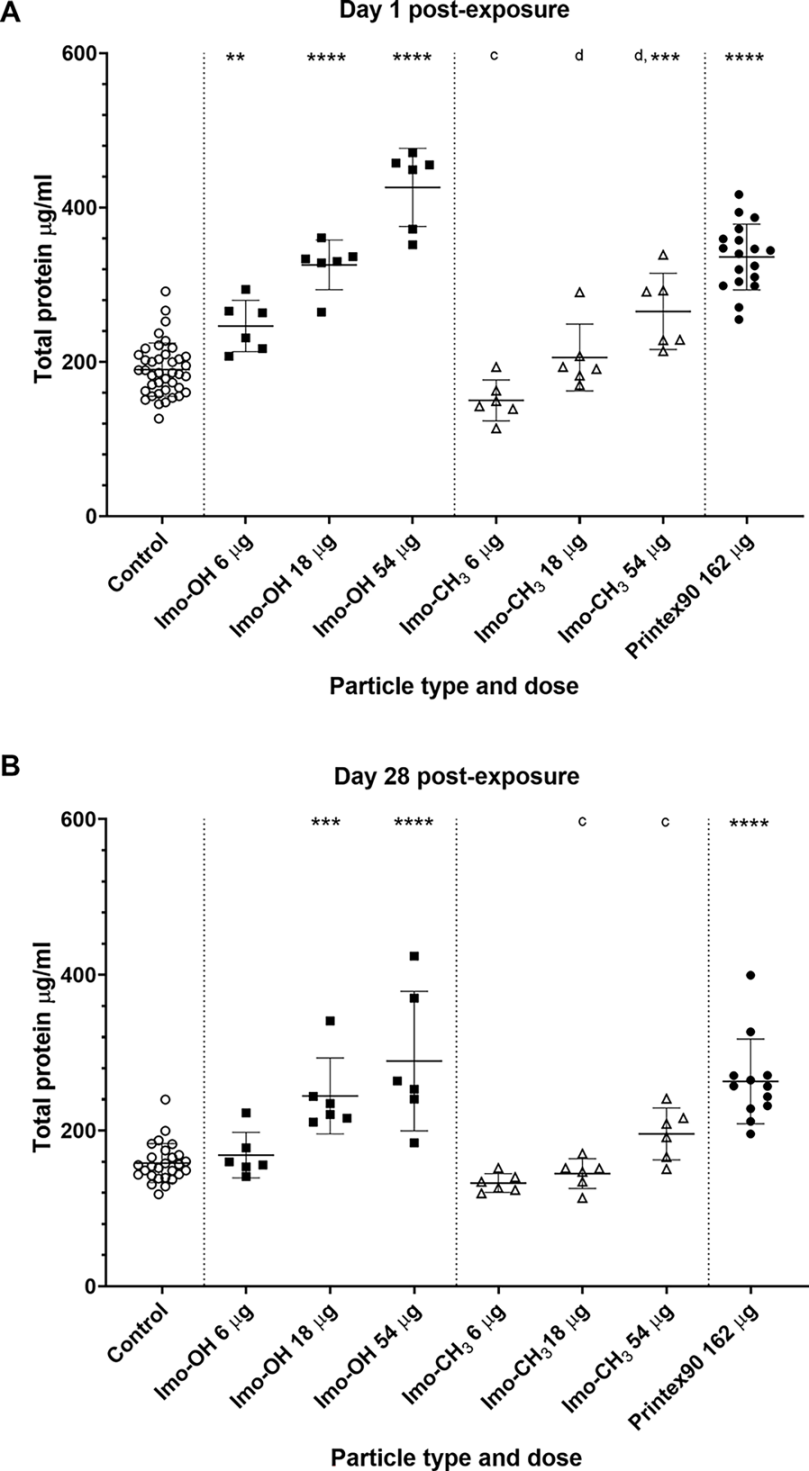
Total protein level in BAL fluid (**A**) 1 and (**B**) 28 days post-exposure to imogolites. Data are shown as individual values, mean ± SD. Stars indicate comparison with the control (Dunnett’s multiple comparison method), and letters indicate comparison to the same dose of Imo-OH (Sidak’s multiple comparison test). Significance levels are as follows: * or a, P ≤ 0.05; ** or b, P ≤ 0.01; *** or c, P ≤ 0.001; **** or d, P ≤ 0.0001

At day 1 post-exposure, Imo-OH elevated total protein in BAL fluid across all doses, whereas Imo-CH_3_ induced a significant effect only at the high dose (Fig. 3A). Overall, the total protein levels at day 28 post-exposure (Fig. 3B) were reduced across all dose groups compared to day 1 post-exposure (Fig. 3A). In the 54 µg Imo‑CH_3_ group, protein levels at day 28 post-exposure have returned to values comparable to the control group, resulting in a lack of significant difference at this time point. However, the percentage decreases observed at 28 post-exposure are similar to those seen at day 1 post-exposure (e.g., 26% decrease for 54 µg Imo‑CH_3_ and 32% for 54 µg Imo‑OH). Notably, Imo-OH showed a sustained response at day 28 post-exposure, unlike Imo-CH_3_ (Fig. 3B). Day 90-post exposure were not analyzed.

The total protein levels was closely mirrored by the neutrophil influx as also previously observed for MWCNTs ([14], Supplemental Fig. S9).

### Acute phase response

**Fig. 4:**
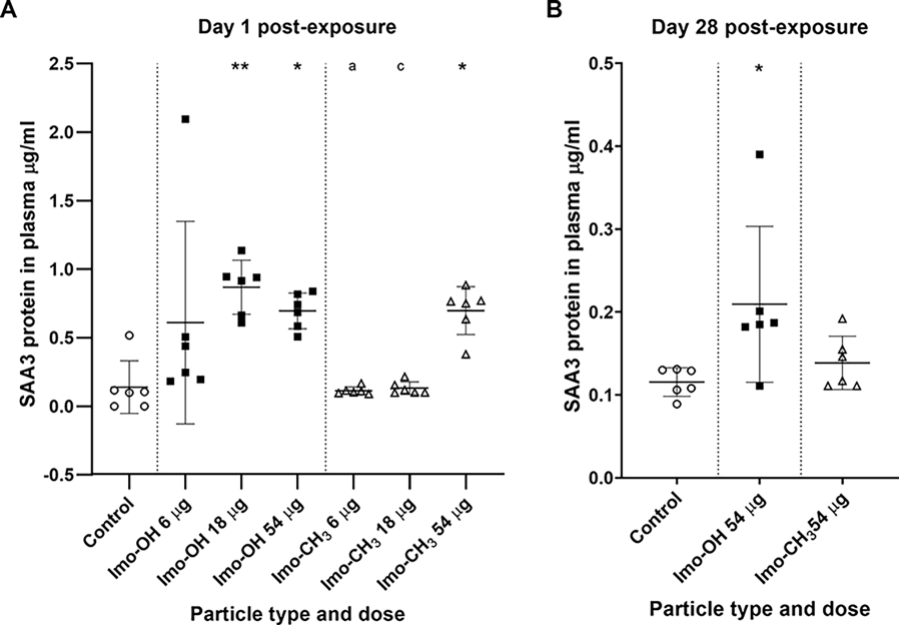
SAA3 protein in plasma (**A**) 1 and (**B**) 28 days post-exposure to imogolites. Data are shown as individual values, mean ± SD. Stars indicate comparison with the control (Dunnett’s multiple comparison method), and letters indicate comparison to the same dose of Imo-OH (Sidak’s multiple comparison test). Significance levels are as follows: * or a, P ≤ 0.05; ** or b, P ≤ 0.01; *** or c, P ≤ 0.001; **** or d, P ≤ 0.0001

At day 1 post-exposure, plasma levels of the acute phase protein SAA3 were significantly elevated for both imogolites at the highest dose (Fig. 4A). Imo-OH also induced a significant increase at the middle dose and maintained a significantly elevated level at the high dose through day 28 post-exposure (Fig. 4B). The elevated SAA3 protein level observed in a single low-dose Imo-OH sample appears to be an outlier (Fig. 4A). We retained this data point for transparency, as we have no technical or biological explanation for its deviation. Due to the high cost of the SAA3 protein assay kit, we prioritized sample inclusion from key experimental groups and excluded Printex90 from this analysis. Based on prior experience, SAA3 plasma protein levels typically return to baseline levels by day 28 following exposure to lower doses [15, 16]. Therefore, at day 28 post-exposure, we focused on the highest doses of Imo-OH and Imo‑CH_3_ to assess the persistence of systemic effects, and the lower doses were excluded from the analysis.

**Fig. 5:**
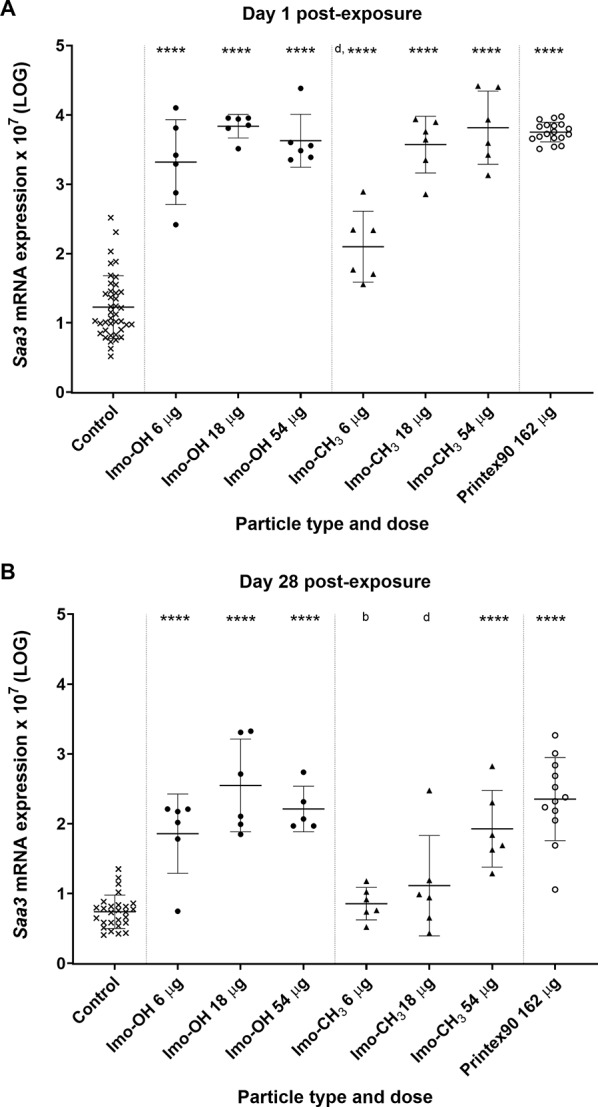
Pulmonary *Saa3* mRNA expression levels (**A**) 1 and (**B**) 28 days post-exposure to imogolites. All values are log transformed and presented as individual values, mean ± SD. Stars indicate comparison with the control (Dunnett’s multiple comparison method), and letters indicate comparison to the same dose of Imo-OH (Sidak’s multiple comparison test). Significance levels are as follows: * or a, P ≤ 0.05; ** or b, P ≤ 0.01; *** or c, P ≤ 0.001; **** or d, P ≤ 0.0001

At the gene expression level, *Saa3* mRNA was significantly increased in lung tissue of mice at day 1 post-exposure by both types of imogolite (Fig. 5A). At day 28 post-exposure, the increase remained statistically significant; however, for Imo-CH_3_, this was observed only at the high dose (Fig. 5B). At day 90 post-exposure, *Saa3* mRNA levels had returned to control levels (Supplemental Fig. S10).

Pearson correlations between neutrophil numbers, pulmonary *Saa3* mRNA, and plasma SAA3 protein levels were analyzed at day 1 post-exposure (Supplemental Fig. S11). Significant correlations were found between neutrophil numbers and *Saa3* mRNA levels (*r* = 0.79, p-value < 0.0001) (Fig. S11A ), between *Saa3* mRNA levels and SAA3 protein levels (*r* = 0.55, p-value 0.0003) (Fig. S11B), and between neutrophil numbers and SAA3 protein levels (*r* = 0.78, p-value < 0.0001) (Fig. S11C), indicating a high level of inter-correlation consistent with previous findings across different types of nanomaterials [51].

**Fig. 6:**
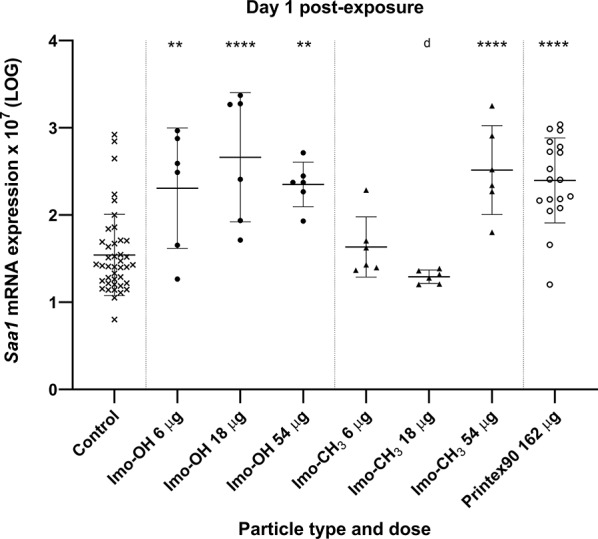
Hepatic *Saa1* mRNA expression levels a day post-exposure to imogolites. All values are log transformed and presented as individual values, mean ± SD. Stars indicate comparison with the control (Dunnett’s multiple comparison method), and letters indicate comparison to the same dose of Imo-OH (Sidak’s multiple comparison test). Significance levels are as follows: * or a, P ≤ 0.05; ** or b, P ≤ 0.01; *** or c, P ≤ 0.001; **** or d, P ≤ 0.0001

In liver tissue, *Saa1* mRNA levels were significantly increased on day 1 post-exposure in response to Imo-OH across all dose levels and in response to the high dose of Imo-CH_3_ (Fig. 6). Gene expression at later time points in liver tissue was not analyzed.

### Histological examination of lung and liver tissue

**Fig. 7:**
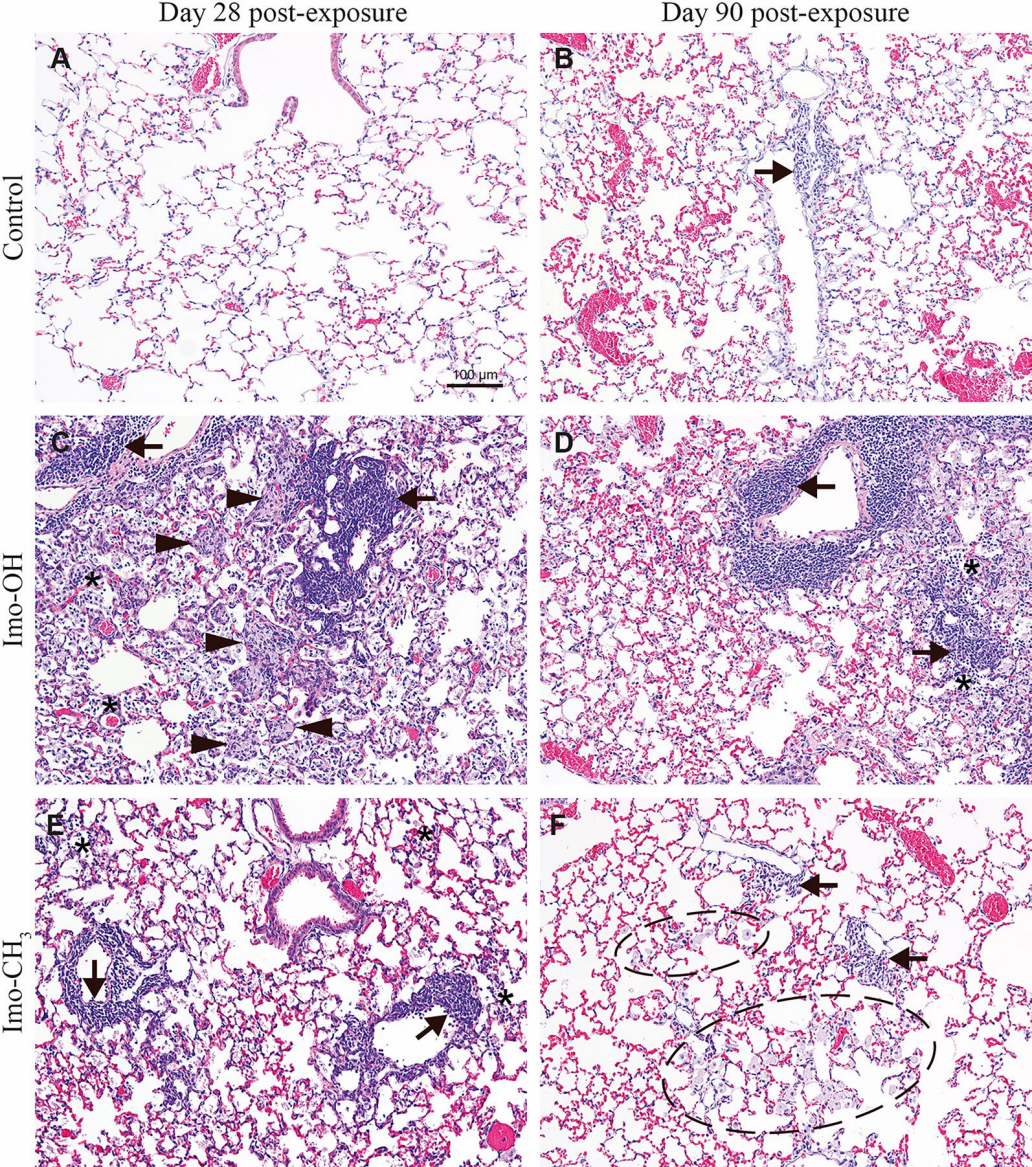
Microscopic changes in the lungs of mice (**A**, **C**, **E**) 28 and (**B**, **D**, **F**) 90 days after a single intratracheal exposure to a vehicle (2% mouse serum in nanopure water; controls) or to Imo-OH or Imo-CH_3_ (54 µg/animal). Asterisks: mononuclear cell infiltrations in alveoli on C, D and E. Arrows: foci of mononuclear cell infiltrations around or at blood vessels in B C, D, E, and F. Arrowheads: aggregates of laden macrophages in C. Ellipse: area of laden macrophages in F.

In the lungs of the mice exposed to Imo-OH and Imo-CH_3_, multifocal mononuclear cell infiltrations at blood vessels, bronchiole or located interstitial or subpleural, laden macrophage aggregates and areas of laden macrophages (examples shown in Supplemental Fig. S12), and areas with debris in alveoli were observed 28 days post-exposure. Overall, the changes were more abundant and severe in mice exposed to Imo-OH group than in mice exposed to Imo-CH_3_ (Table 2 and Supplemental Table S4). Figure 7 depicts examples of the microscopic changes in the lungs of mice after exposure to a high dose of imogolites in comparison to control mice. There were no changes in the control animals on day 28 post-exposure (Fig. 7A) and minimal to mild mononuclear cell infiltrations were sporadically observed in the control animals on day 90 post-exposure (Fig. 7B). The changes appeared more severe in mice exposed to Imo-OH at both time points post-exposure (Fig. 7C and D) compared to mice exposed to Imo-CH_3_ (Fig. 7E and F). The aggregates of laden macrophages in mice exposed to Imo-OH often had a differentiated appearance with no clear foamy structure of the cytoplasm while in mice exposed to Imo-CH_3_ their foamy appearance was preserved. The macrophage aggregates and areas were localized either at the bronchioalveolar junctions, alveolar ducts or in alveoli.

**Table 2:** Type and incidence of microscopic changes in the lungs of mice 28 and 90 days after a single intratracheal exposure to a vehicle (2% mouse serum in nanopure water; controls) or to Imo-OH, Imo-CH_3_ (54 µg/animal)

Day post exposure Type of change	Day 28			Day 90		
	Control *n* = 6	Imo-OH *n* = 5	Imo-CH_3_ *n* = 5	Control *n* = 6	Imo-OH *n* = 5	Imo-CH_3_ *n* = 5
Mononuclear cell infiltration^B^ peribronchial, perivascular, interstitial or subpleural (focal or multifocal)	0/6^A^	5/5** 0/0/0/0/5^F^	5/5** 0/0/1/2/2	3/6 0/1/1/1/0	5/5 0/0/1/0/4	5/5 0/2/1/0/2
Mononuclear cell infiltration in single alveoli, focal	0/6	5/5** 0/1/1/0/3	4/5 0/0/2/1/1	1/6 1/0/0/0/0	5/5* 0/2/3/0/0	3/5 3/0/0/0/0
Single, laden alveolar macrophages	0/6	5/5** 1/1/1/1/1	5/5** 3/1/0/1/0	0/6	5/5** 2/3/1/0/0	5/5** 5/0/0/0/0
Area of laden alveolar macrophages^C^ (focal or multifocal)	0/6	5/5** 1/0/3/0/1	5/5** 0/0/2/3/0	0/6	5/5* 1/0/2/1/1	4/5 0/1/1/2/0
Small aggregates of laden macrophages ^D^ (focal or multifocal)	0/6	4/5 1/3/0/0/0	3/5 1/2/0/0/0	0/6	3/5 1/2/0/0/0	0/5
Large aggregates of laden macrophages ^D^ (focal or multifocal corresponding to severity grade G3-G5)	0/6	5/5** 0/0/0/0/5	1/5 0/0/1/0/0	0/6	3/5 0/0/2/1/0	0/5
Debris in alveoli	0/6	5/5** 0/1/0/0/4	5/5** 2/0/0/1/2	0/6	5/5** 2/0/0/0/3	3/5 1/0/0/1/1
Hypertrophy/hyperplasia of type II pneumocytes	0/6	4/5 2/1/1/0/0	0/5	0/6	1/5 1/0/0/0	0/5
Desquamation of bronchiole epithelium^E^	0/6	5/5** 5/0/0/0/0	0/5	0/6	3/5 3/0/0/0/0	2/5 2/0/0/0/0

* *p* > 0.05, ** *p* < 0.01 Fisher exact test. The data on incidence and severity originate from the whole lung of each animal i.e. from the left, right cranial, right middle, right accessory and right caudal lobes

^A^ Incidence of each change is expressed by the number of animals with a given change of a total animals examined in the group

^B^ Mononuclear cell infiltrations: an area of lung tissue where the density of mononuclear cells is increased compared to the background of the surrounding, so that the mononuclear cells collection has a shape and size. Only the infiltrations of more than 20 cells were counted. The infiltrations consist usually of lymphocytes but other inflammatory cells like neutrophils or eosinophils can also be present in the infiltration. The infiltrations are observed near blood vessels, or bronchioles or alveolar ducts, interstitial or subpleural

^C^ Area of laden macrophages: an area of tissue where the density of macrophages is higher than background but the laden macrophages are situated not close to each other

^D^ Laden macrophage aggregates: groups of alveolar laden macrophages at one site close to each other. The aggregates of severity grade 1 to 2 are considered as small and the aggregates of severity grades from 3 to 5 are considered as big

^E^ This change was not recorded, when there was an indication of a tangential cut of bronchioles. A tangential cut can result in an artifact resembling a desquamation of bronchiolar epithelium

^F^ Severity of a given change was evaluated in a semi-quantitative way using a five grade scoring system. Grade 1: minimal/very few/very small; grade 2: mild/few/small; grade 3: moderate/moderate number/moderate size; grade 4: marked/many/large; grade 5: massive/extensive number/extensive size. Results for severity are presented as a number of animals per group for which a given change was assigned to a certain grade either G1/G2/G3/G4/G5. When a given change was seen in several sites of the lungs, each focus was assigned a severity grade, and the highest severity grade was chosen to represent the animal in this table. For example, the severity reported as 0/1/2/0/0 means that 1 animal in the group had a given change of the maximum severity graded as G2 (small) and 2 animals had a given change of the maximum severity graded as G3 (moderate size)

On day 90 post-exposure the mononuclear cell infiltrations, and areas of laden macrophages and debris were still present in both imogolite exposed groups. These changes appeared more severe in the Imo-OH group compared to the Imo-CH_3_ group (Fig. 7D and F). The aggregates of laden macrophages were only seen in the Imo-OH group (Fig. 7D).

**Fig. 8:**
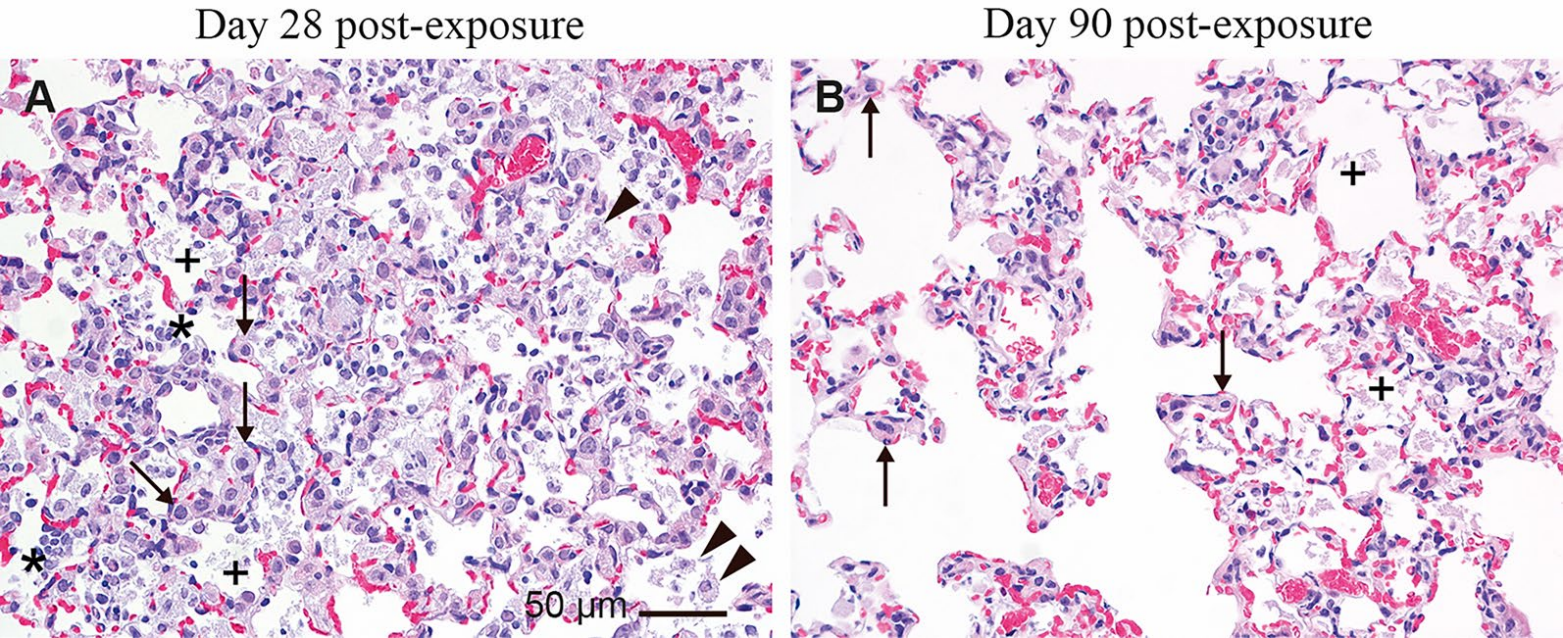
Imo-OH, **A**: day 28 post exposure. Hypertrophy/hyperplasia of type II pneumocytes (arrows) and increased presence of laden alveolar macrophages (arrowheads) variably associated with mononuclear inflammatory cell infiltrate (asterisks). **B**: day 90 post exposure. Some of type II pneumocytes were slightly larger than normal (long arrows). Debris (+) in alveoli and alveolar ducts

Hypertrophy/hyperplasia of type II pneumocytes of minimal to moderate severity was observed in Imo-OH group at day 28 post-exposure (Fig. 8A; Table 2). Some of the type II pneumocytes appeared slightly larger than in controls at day 90 post-exposure (Fig. 8B). Hypertrophy/hyperplasia was not observed in the control groups or in mice exposed to Imo-CH_3_ at any time points.

Microscopic examination of the liver samples from imogolite-exposed mice 28 and 90 days post-exposure did not reveal any morphological differences from controls in the type, incidence and severity of the observed changes (Supplemental Table S5 and S6).

### Comparisons of imogolites with other nanotubes

**Fig. 9:**
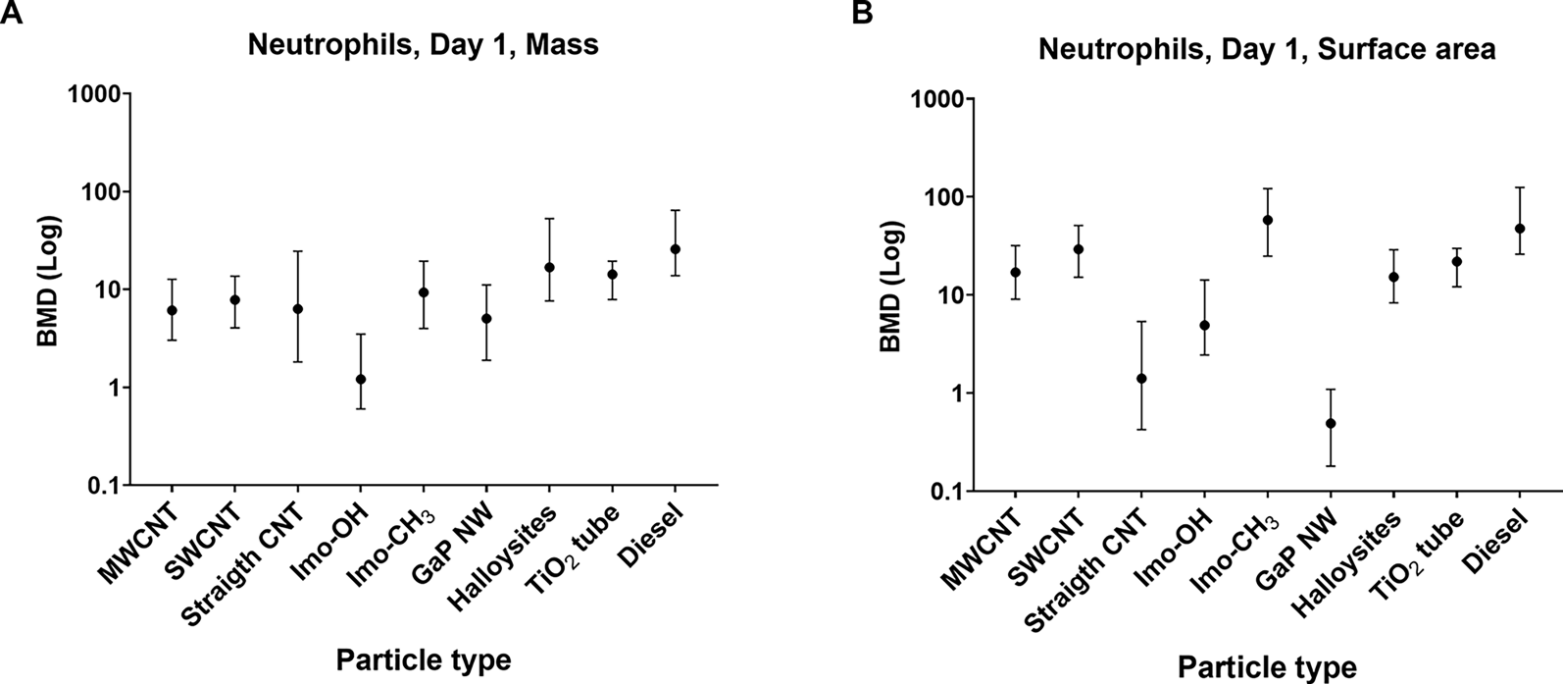
Benchmark dose (BMD) modelling of neutrophil influx 1 day post-exposure for the studied imogolites, entangled MWCNTs (n=20), SWCNTs (n=7), straight and long MWCNTs (n=2), straight and long GaP NWs (n=1), straight and short Halloysite fibers (n=2), TiO2 tube (n=1) and diesel engine exhaust particles (n=7). The mass dose levels (A), and the surface area dose levels (B) were modelled separately

The inflammatory potential of imogolites was compared with other high-aspect-nanomaterials (HARNs) using benchmark dose (BMD) modelling of neutrophil influx on day 1 (Fig. 9). The BMD for Imo-OH-induced inflammation was compared to BMD values for previously published HARNs including entangled MWCNTs, SWCNTs, long and straight MWCNTs [16, 17], long and straight GaP nanowires [52], short and straight nanofibers such as halloysite fibers [53] and TiO_2_ nanotubes [54]), as well as diesel exhaust particles, that were included in the analysis as a benchmark material with known toxicity [55–57]. The mass-based BMD for Imo-OH was lower than that of other HARNs, whereas Imo-CH_3_ BMD values were comparable to that of other HARNs and lower than the BMD for diesel exhaust particles. For the surface area-based BMD, Imo-OH had the lowest BMD, along with straight CNTs and GaP nanowires.

**Fig. 10:**
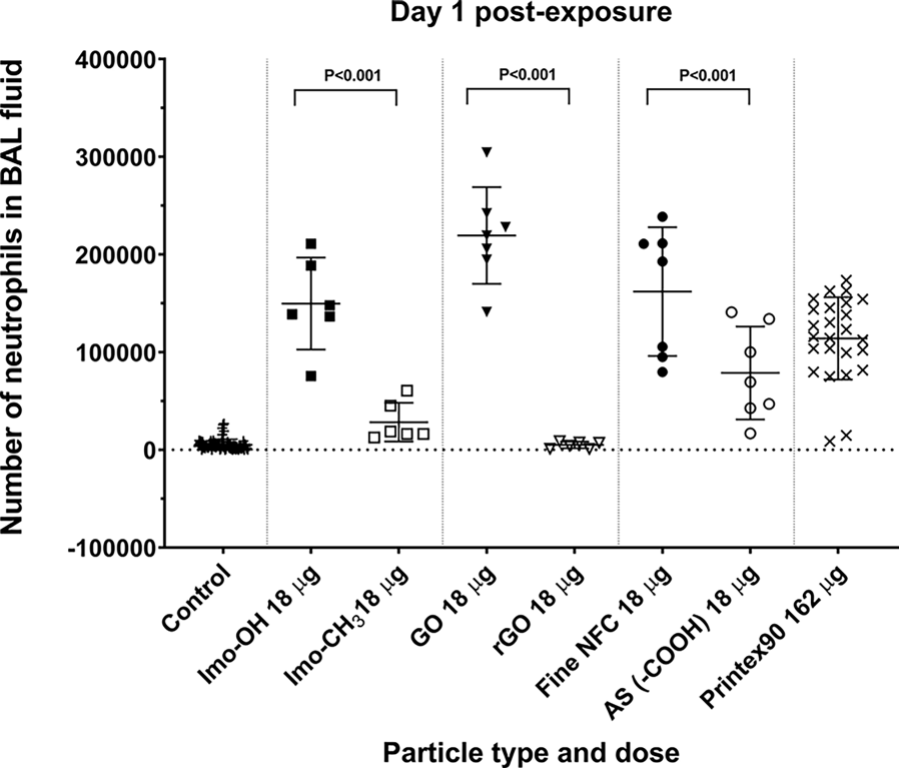
Comparison of neutrophil influx in mice lungs after intratracheal instillation to the same mass dose levels of imogolites, graphene oxides and nanocelluloses at day 1 post-exposure. Original data for graphene oxide (GO) and reduced graphene oxide (rGO) is published in Bengtson et al. 2017 and nanofibrillated cellulose (NFC) and carboxylated cellulose (AS (-COOH)) is published in Hadrup et al. 2019

Previously, we investigated the inflammatory effects of highly hydroxylated materials in vivo, comparing them to corresponding functionalized materials without free OH groups using methodologies similar to those in the present study [58, 59]. A comparison of neutrophil influx at day 1 post-exposure, at equivalent mass doses, revealed a significant reduction when mice were exposed to reduced graphene oxide (rGO) versus graphene oxide (GO) and carboxylated nanofibrillated cellulose (AS (-COOH)) versus unmodified nanofibrillated cellulose (Fine NFC) (Fig. 10). Notably, GO and Fine NFC were highly hydroxylated, whereas rGO and AS (-COOH) were not.

## Discussion

In this study, we assessed the pulmonary toxicity of imogolites with a focus on inflammatory responses and systemic acute phase response. Pulmonary inflammation and systemic responses are critical endpoints linked to long-term health effects such as fibrosis, cancer, and cardiovascular disease [60, 61].

Although inhalation studies are the gold standard of toxicity testing of inhalation exposure, intratracheal instillation, used in this study to expose mice to imogolites, is an established method for hazard comparison. It allows precise control of the dose deposited in the lungs and ensures even distribution across lung lobes, as demonstrated in previous studies [14, 62, 63]. For imogolites, van den Brule et al. demonstrated that intratracheal instillation is a powerful method to study the lung toxicity of nanometer long Ge-based imogolites [26]. Moreover, the correspondence between inhalation and instillation exposures as previously shown for CNT-induced neutrophil influx [64] and lung distribution of metal oxides [63] supports the relevance of this approach for toxicity studies.

Both imogolite variants triggered a robust acute inflammatory response in the lungs, characterized by substantial neutrophil influx (Fig. 2). However, only Imo-OH exposure resulted in persistently elevated neutrophil levels at later time points. The increased neutrophil influx is associated to elevated protein levels in BAL fluid (Fig. 3). Mechanistically, the increased protein levels can be attributed to damage to the alveolar-capillary barrier, allowing protein leakage into the lungs; epithelial cell damage, leading to the release of intracellular proteins; and cytokine secretion by immune cells, which exacerbates tissue injury and increases vascular permeability. These findings align with previous studies demonstrating strong correlations between neutrophil influx and protein levels after airway exposure to nanomaterials [14, 63]. Similarly, Van den Brule et al. showed a correlated increase of neutrophil influx and protein level for Ge-based imogolite although not hightlighted by the authors [26]. Additionally, the elevated protein levels observed in this study are consistent with AOP173, where increased membrane permeability is recognized as a key event [65]. While AOP173 specifically describes membrane permeability changes, the presence of elevated proteins in BAL fluid can be considered a supporting indicator of this key event. Increased membrane permeability is a critical early step in the development of fibrosis, as described in AOP173, which outlines how inhalation of fibrous materials such as carbon nanotubes (CNTs) may induce fibrosis [65].

The systemic acute phase response, assessed in terms of SAA3 plasma protein levels, showed significant increases for both imogolites at the highest dose on day 1 post-exposure (Fig. 4). Notably, Imo-OH also caused a significant elevation at the middle dose on day 1 and sustained increased SAA3 levels through day 28, indicating a prolonged systemic response. This pattern reflects that while *Saa3* gene expression at day 28 was significantly elevated, the *Saa3* mRNA levels were much lower than at day 1 post-exposure (Fig. 5). We have previously shown that highly increased Saa3 mRNA levels are required before increased SAA3 protein levels may be detected in plasma [51]. The transient elevation of *Saa3* mRNA levels at Day 1 and 28 reflects the acute phase nature of the response. These increases highlight an early and intermediate transcriptional activation following exposure, which resolves by day 90 post-exposure, indicating recovery. This pattern underscores the utility of *Saa3* mRNA levels as a sensitive marker for early and subacute systemic responses to nanomaterial exposure.

Systemic effects were also confirmed by the upregulation of hepatic *Saa1* mRNA 1 day after pulmonary exposure (Fig. 6), consistent with previous studies on various nanomaterials [15, 37, 51, 54, 66–68]. Hepatic *Saa1* upregulation may be triggered either by direct translocation of nanomaterials to the liver [20, 52, 68, 69] or indirectly via cytokines and acute phase mediators released from the inflamed lung [15, 70]. The transient nature of this response is well-documented: the hepatic acute phase response typically peaks around 1 day post-exposure and resolves relatively quickly, whereas the pulmonary response persists [15]. Accordingly, *Saa1* expression was measured only at the early post-exposure time point in this study. Despite evidence of a hepatic acute phase response, no histopathological liver changes were observed 28 days post-exposure. These findings support the liver’s role as a secondary target in the systemic response to inhaled nanomaterials without progression to overt pathology, as acute phase reactions are functional and do not necessarily lead to structural damage.

The acute phase response is particularly concerning due to its systemic implications, including an increased risk of cardiovascular disease [18, 19]. Elevated plasma SAA3 levels promotes plaque formation, as demonstrated in susceptible mouse models [70–72], linking particle and fiber-induced pulmonary acute phase response to atherosclerosis and thereby cardiovascular disease. The systemic effects observed in this study highlight the potential long-term health risks of sustained pulmonary inflammation and acute phase responses following exposure to imogolites as observed for both Imo-OH and Imo-CH_3_.

The inflammation observed in this study is consistent with the common understanding of pulmonary responses to particulate exposure. Pulmonary inflammation represents an early key event associated with adverse health outcomes, including cancer, fibrosis, and chronic obstructive pulmonary disease [61, 71, 72]. A hallmark of this inflammatory response is the influx of neutrophils into the lung, which, in this study, correlated with elevated levels of the acute phase protein SAA3 in plasma and *Saa3* mRNA expression in lung tissue (Fig. S6). Such correlations have been observed across exposure methods (inhalation and instillation), post-exposure time points, and nanomaterial types [15, 51, 54, 58, 59, 67, 73, 74].

These toxicological findings are confirmed by the histopathological evaluation of lung tissue, where all changes, both in terms of incidence and severity score, appeared significantly more predominant in the Imo-OH group compared to the Imo-CH_3_ group at both day 28 and day 90 post-exposure (Fig. 7). Interestingly, hypertrophy/hyperplasia of alveolar epithelial type II cells was observed in the lung after exposure to Imo-OH but not to Imo-CH_3_, further highlighting the toxicity differences between the two imogolites (Fig. 8).

The inflammatory potential of imogolites was assessed in comparison to other HARN using benchmark dose (BMD) modeling (Fig. 9). Overall, imogolites elicited an acute inflammatory response that was broadly comparable to the BMDs observed for other HARNs, including SWCNTs, short and thin MWCNT, long and straight MWCNTs, and other HARNs included in the analysis. This indicates that imogolites induce a similar level of inflammatory response as these materials. Notably, Imo-OH exhibited the lowest BMD by mass, and when assessed by surface area, it ranked among the lowest, alongside straight CNTs and GaP nanowires. Diesel engine combustion particles were included as a benchmark material because of their well-documented toxicity and occupational exposure limits for diesel engine exhaust in many countries, based on chronic inhalation studies in rats and human epidemiological data [75–77]. Previous studies from our group have reported inflammatory, acute-phase response, and genotoxic effects following pulmonary exposure in mice (Bendtsen et al. 2019; Bendtsen et al. 2020; Kyjovska et al. 2015). In the present BMD analysis, diesel engine combustion particles demonstrated a higher BMD compared to all HARNs tested. This suggests that HARNs – including imogolites, and particularly Imo-OH − have a stronger inflammatory potency than diesel combustion particles.

The observed differences in toxicity between Imo-OH and Imo-CH_3_ are noteworthy. Imo-OH induced stronger and more persistent inflammatory responses and systemic effects compared to Imo-CH_3_.

This distinction cannot be explained by differences in BET surface area, as normalization (407 m²/g for Imo-OH vs. 625 m²/g for Imo-CH_3_) would only amplify the disparity in their toxicity. Moreover, as underlined by Dhaini et al., the proportion of bundles can strongly influence the BET specific surface and as a consequence the accessible surface of the imogolite, leading to underestimation of the specific surface area of Imo-CH_3_. This underlines the need for methods able to give an absolute value of the specific surfaces accessible in the given experimental conditions. Thus, Imo-CH_3_ induces less inflammation than Imo-OH despite the larger specific surface area, which may be underestimated due to agglomeration. Another important point is the amount of residual small particles (protoimogolite and allophane) in equilibrium with the nanotubes. For Imo-OH, the proto-imogolites are well dispersed, while for Imo-CH_3_, the proto-imogolites are in majority stuck on the external surface of the nanotube with probably a lower reactivity. Going from -OH to -CH_3_ in the inner cavity also impacts the length distribution, as the studied samples exhibit a longer average length for Imo-OH (ca. 600 nm) than for Imo-CH_3_ (ca. 300 nm). However, both Imo-OH and Imo-CH_3_ are short as compared to fiber paradigm-predicted toxicity, which is relevant for fibers longer than 3–5 μm [78].

Another strong difference is the variation in the chemical composition, particularly the functional groups present on their surfaces. Imo-OH has a higher degree of hydroxylation, but at the inner surface of the tube, so it is unclear how this contributes to its increased toxicity, in particular because the inner diameter is less than 2 nm and the Imo-OH inner cavity contains confined structured water strongly linked by H-bond, while Imo-CH_3_ contains weakly H-bonded water molecules like in CNT [79]. However, the link between hydroxylation and toxicity is supported by other studies as illustrated in Fig. 10. A highly hydroxylated graphene oxide (GO) induced significantly stronger acute pulmonary inflammation and a more robust acute phase response than reduced graphene oxide (rGO), where the surface hydroxylation was significantly reduced [58]. Similarly, carboxylated nanofibrillated cellulose were shown to reduce neutrophil influx and systemic acute phase responses compared to unmodified nanofibrillated cellulose, suggesting that carboxylation of the OH groups reduces the nanofibrillated cellulose-induced toxicity [59]. These findings indicate that modifying the surface chemistry of highly hydroxylated nanomaterials could be an effective strategy for reducing their toxicity while maintaining their functional properties, representing a safe and sustainable by design (SSbD framework) approach. However, as stated previously, the hydroxyl part of the inner surface of the tube is not easily accessible, indicating an indirect effect of going from -OH to -CH_3_ causing the toxicity differences. Beyond the difference in bundle formation, in the presence of isolated proto-imogolite, changing OH by CH_3_ also modifies the length of the nanotube and the direct band gap (from 5.83 ± 0.3 eV to 5.4 ± 0.2 eV, respectively [7]. This variation could have consequences on the production of ROS and, consequently, influence toxicity. Liu et al. have shown that toxicity is associated with ROS production for short nanotubes whereas longer nanotubes exhibit toxicity through a different mechanism [24]. A recently published QSAR model can predict neutrophil influx based on physicochemical properties and modeling using materials with similar characteristics [80]. It would be highly relevant to assess whether this modeling approach also identifies surface hydroxylation as a pro-inflammatory property.

Finally, the application of traditional, state-of-the-art cell culture analyses for comparing further the indicated toxicity differences of the two imogolite forms in vitro were unexpectedly unsuccessful as the imogolites solidified the cell culture medium (unpublished results).

In conclusion, this study highlights significant differences in the toxicity profiles of Imo-OH and Imo-CH_3_ following pulmonary exposure in mice, demonstrating that blocking hydroxyl groups may serve as a safe-by-design strategy to reduce imogolite-induced inflammation and acute phase response, even when the reduced toxicity results from indirect effects not directly related to hydroxyl interactions with biological media. Importantly, indirect effects arising from changing OH with CH_3_ within the inner cavity, such as changes in length, bundle formation, the presence of small by-product particles, or alterations in band gap are also critical factors to consider when predicting the evolution of toxicity. However, benchmark comparisons indicate that, upon intratracheal instillation, imogolites exhibit a high inflammatory potential, comparable to other HARNs. These findings emphasize the importance of carefully considering the physicochemical properties of nanomaterials in the development of safer products, particularly in occupational settings where pulmonary exposure risks are elevated.

## Supplementary Information

Supplementary Material 1

## Data Availability

No datasets were generated or analysed during the current study.
