# Supplementary material for: Imogolite nanotube modifications impact pulmonary toxicity in mice: implications for safe and sustainable by design (SSbD)
Source: J Nanobiotechnology. 2025 Aug 18;23:571. doi: 10.1186/s12951-025-03647-w (PMC12359848; doi:10.1186/s12951-025-03647-w)
Supplement: Supplementary file 1 — Supplementary Material 1 [file 12951_2025_3647_MOESM1_ESM.docx]

**Synthesis protocol of Imo-OH and Imo-CH_3_ and Characterization Protocols**

**Synthesis of Imo-OH:** 2 mM aluminium (AlCl_3_, 6H_2_O) solution is first prepared trough the slow addition of the powder in water with discontinuous stirring. Tetraethoxysilane is then slowly added under vigorous stirring and maintained under stirring for 30 min at room temperature. After, a 0.1M sodium hydroxide solution is added at 0.1 ml/min to attain an OH/Al ratio of 2 and stirred until the disappearance of turbidity. The solution is kept in a closed recipient at 90°C for 5 days. After cooling, the solution is concentrated by ultrafiltration system (Polymem, Toulouse, France) with a 300kDa persulfon membrane. Then, the suspension is purified by centrifugation at 9000 rpm to remove the aluminium hydroxide before a final dialysis against water with 8 - 10kD membrane (until the conductivity of 2µS.cm^-1^ is obtained).

**Synthesis of Imo-CH_3_:** The protocol is adapted from Bottero et al (Bottero *et al.* 2011). Aluminium-tri-sec butoxide is added to a HCl solution (75mM) containing trimethoxymethylsilane (TMMS), the proportion are adapted to attain a Al:HCl:Si molar ratio equal to 1.8:1:1. Vigorous stirring is maintained at room temperature during 1 hour to decrease the turbidity. The solution is then kept in a closed vessel at 90°C during 5 days. After cooling, the solution is centrifuged at 9000rpm to eliminate the aluminium hydroxides. Then the solution is dialyzed against water with 8 - 10kD membrane (until the conductivity of 2µS.cm^-1^ is obtained). The solution is then dried in a spray drying system (Buchi B290) to obtain powder. The drying parameters are fixed with an inlet temperature of 170°C, a suction of 100%, a pumping of 30% and a drying gas flow rate of 473 l/h.

**Characterization of imogolites**

InfraRed (IR) was performed on a Bruker Tensor 27 FTIR (Fourier Transform infrared) controlled by OPUS software (version 7.5). IR was performed on dried powder dispersed in KBr.

XPS was performed on dried powder with Kratos Axis Ultra DLD spectrometer with monochromatic Al Kα excitation (1486.7 eV) at 150 W and a charge compensation system. The individual core level spectra were collected at an analyzer pass energy of 20 eV. Calibration was performed using the standard adventitious Al 2p peak at 74.4 eV. XPS spectra were analyzed using a nonlinear Shirley-type background. The core peaks and areas were spotted by a weighted least-squares fitting method using Lorentzian line shapes.

The imogolite suspensions were observed by cryogenic transmission electron microscopy (cryoTEM) on a JEOL 2010 FEG microscope operated at 200 kV. The thin vitreous ice film is formed after dropping the suspension on copper grids covered with a holey carbon film (Quantifoil R2/2) previously treated with a plasma glow discharge (5 mA, 30 s) and using a Vitrobot apparatus (FEI Company). Observations were conducted at low temperature (-180°C). The images were acquired with a Gatan K2 direct detection camera. Typically, a stack of 20 dose-fractionated images (0.2 s acquisition/frame with a maximum dose of 20 e^−^/pixel/second) was recorded and then aligned to compensate for specimen motion, drift and irradiation damage with the Gatan Microscopy Suite DigitalMicrograph.

Small Angle X-ray scattering (SAXS) was acquired at the SWAXS Laboratory (https://iramis.cea.fr/nimbe/lions/swaxs-lab/). Acquisitions were obtained on XEUSS 2.0 instrument (XENOCS) equipped with a Cu microfocus X-ray sources collimated with scatter-less slit technology and a Pilatus 1M detector (Dectris). The calibration were done though PyFai and PySAXS open source software (https://pypi.org/project/pySAXS/) using silver behenate for the detector to sample distance (40 cm) and the direct beam measurement for the normalization of detector count into the differential cross section (Taché *et al.* 2016). The scattered intensity was plotted in absolute units (cm^-1^) versus the wave vector q = (4π/λ) sin (2θ) expressed in function of the scattered angle 2θ. The sample were analyzed on a 1.5 mm borosilicate capillary (Hilgenberg) under secondary vacuum to minimize the scattering from air, with an acquisition time of 1800s.

The specific surfaces were measured on MICROMERITICS 3-Flex apparatus from N2 adsorption. Imo-OH and Imo-CH_3_ were prior degassing under vacuum at 275°C during 4 h and at 150°C during 4 h respectively. Specific surfaces are extracted from a BET surface area plot.

The distribution in length of the Imo-OH nanotubes was extracted by AFM analysis after deposition on a silicon wafer. The samples were investigated with a Veeco Dimension 3100 AFM equipped with a Nanoscope IIIa controller.

For Imo-CH_3_, the length distribution was obtained with image J treatment of TEM images issued from a FEI CM12 TEM microscope after deposition of the suspension on a copper grid. The TEM images are registered at 80 kV with a Gatan CCD camera, a spot size of 4 and a magnification of x2650-x100000.

**References:**

Bottero *et al.* 2011. Synthesis and characterization of hybrid organic/inorganic nanotubes of the imogolite type and their behaviour towards methane adsorption. Phys Chem Chem Phys 13(2):744-750, doi:10.1039/c0cp00438c.

Taché *et al.* 2016. MOMAC: a SAXS/WAXS laboratory instrument dedicated to nanomaterials, J. Appl. Cryst. 49, 1624–1631, doi:10.1107/ S1600576716012127.

| 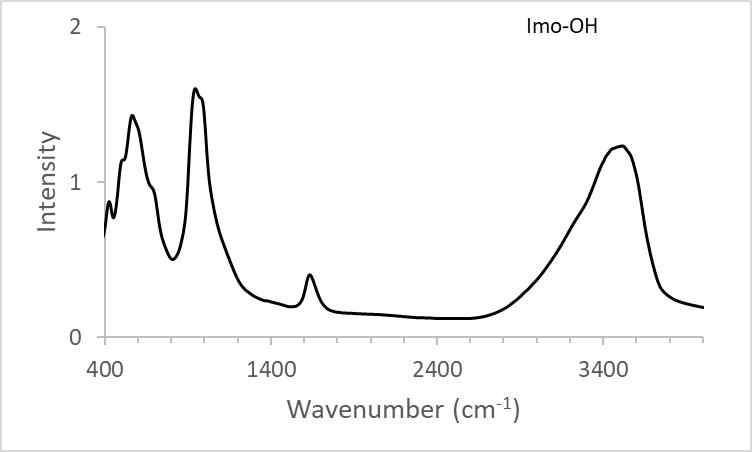 | 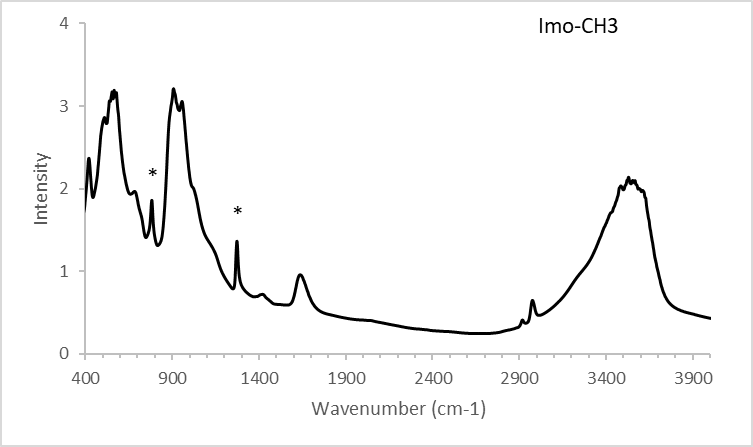 |
| --- | --- |

**Fig. S1.** IR-spectra of synthesized imogolites (left) Imo-OH and (rigth) Imo-CH_3_ using KBr techniques. (*) identified the Si-C and the Si-CH_3_ bounds for Imo-CH_3_. The spectra are typical of Imo-OH and Imo-CH_3_ respectively in agreement with the IR-characterization described in Liao et al. Appl Clay Sci 2018, 164:58-67 and in Picot et al. 2016, Faraday Discuss, 191:391-406.

| 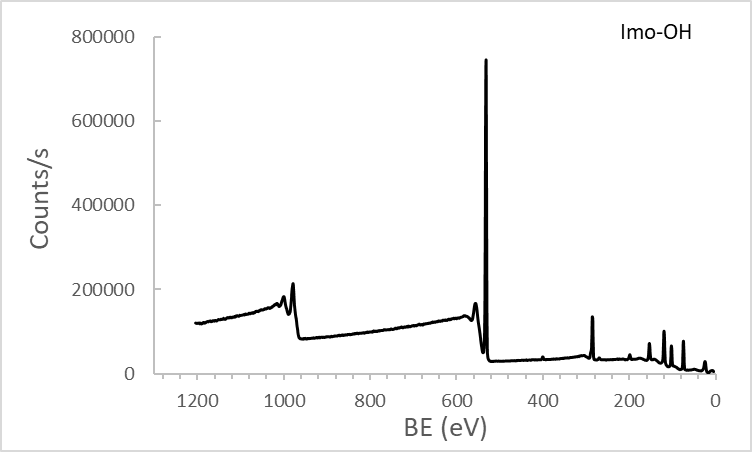 | 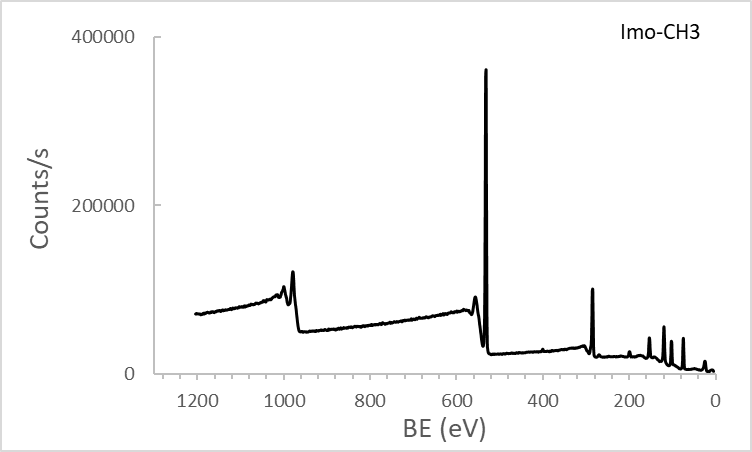 |
| --- | --- |

**Fig. S2.** XPS spectra survey scan for Imo-OH (left) and Imo-CH_3_ (right) powder, the spectra are typical of Imo-OH and Imo-CH_3_ as described in Pignie et al. 2021, Nanoscale, 13(46):19650-19662.

| 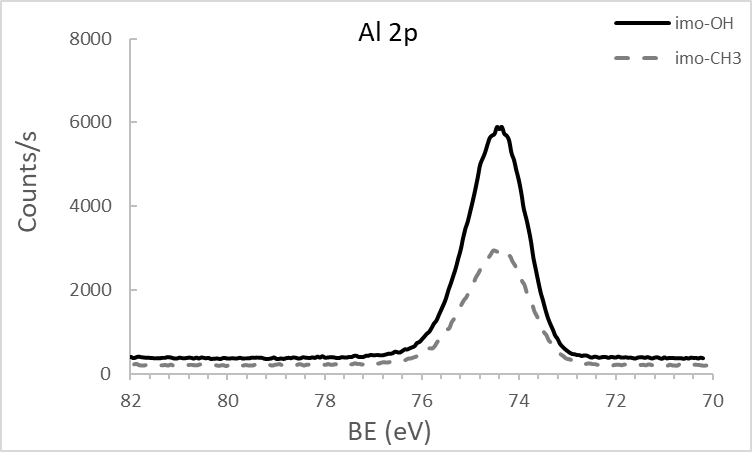 | 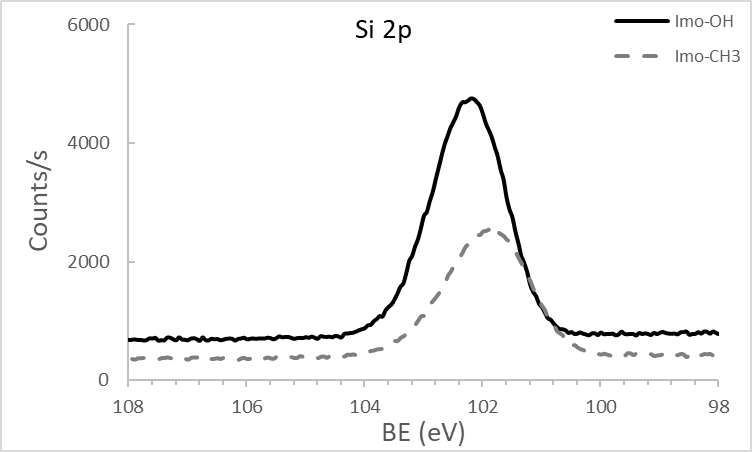 |
| --- | --- |
| 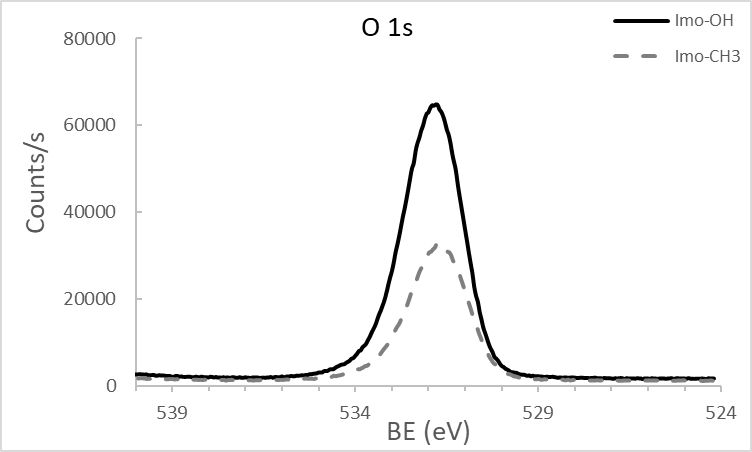 | 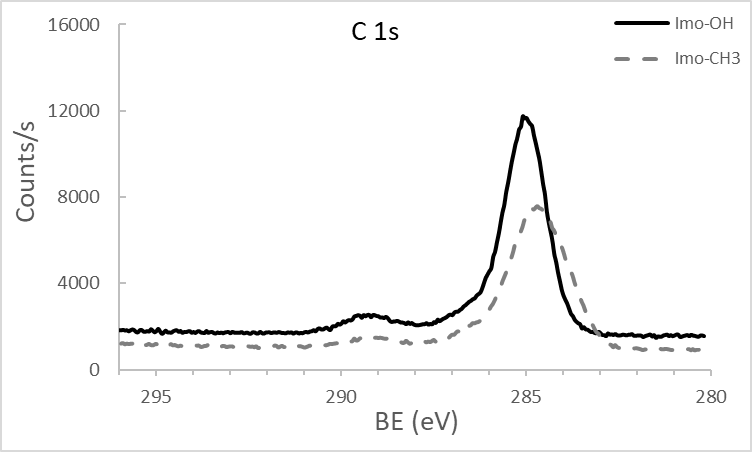 |

**Fig. S3.** Core level scan spectra for Imo-OH (full line) and Imo-CH_3_ (dashed line) powders for Al 2p; Si 2p; O 1s and C 1s. Energy calibration was done on Al 2p = 74.4 eV to extract the Si/Al ratio. Si 2p and C 1s clearly exhibits a difference between Imo-OH and Imo-CH_3_ detailed hereafter in Fig. S4 and S5.

| 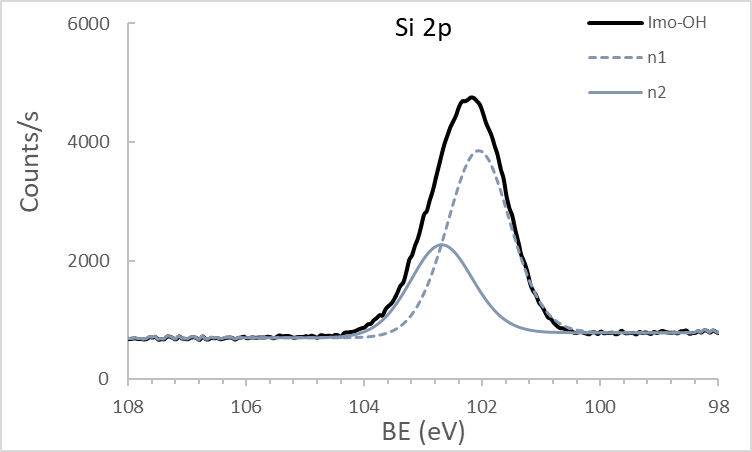 | 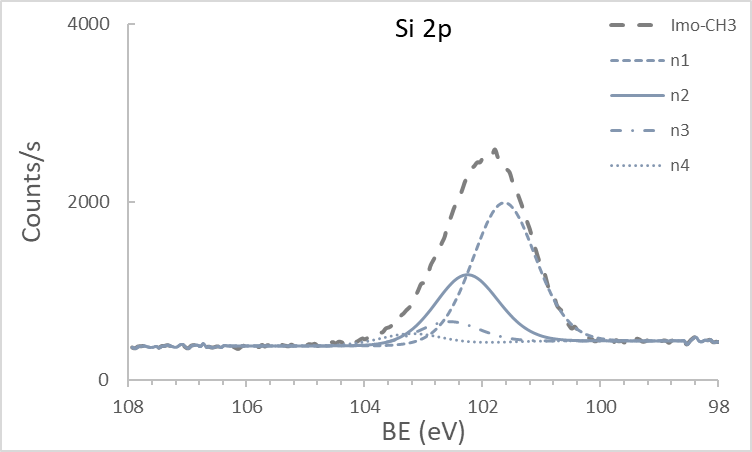 |
| --- | --- |

**Fig. S4.** Different contributions (ni) to Si 2p core level spectra for Imo-OH (left) and Imo-CH_3_ (right) powders with the typical contributions for each and in particular for Imo-CH_3_ (at 101.7 eV as mentioned in Zanzottera et al. 2012, J. Phys. Chem. C, 116, 7499-7506. However for Imogolite, attribution should be taken with caution due to the wall polarization as described in Pignie et al. 2021, Nanoscale, 13(46):19650-19662.

| 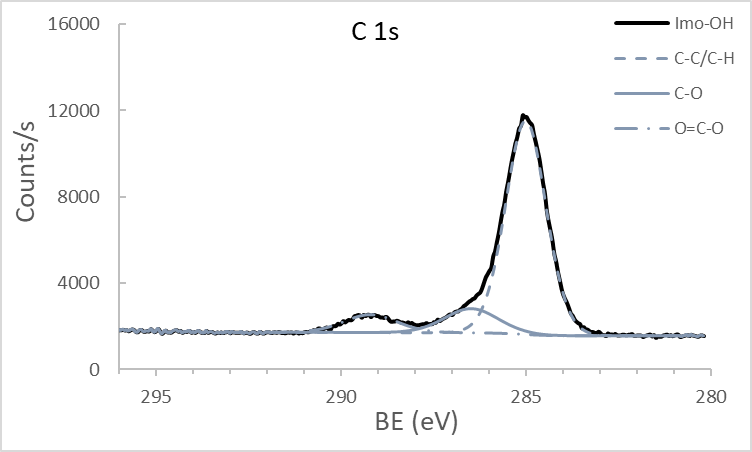 | 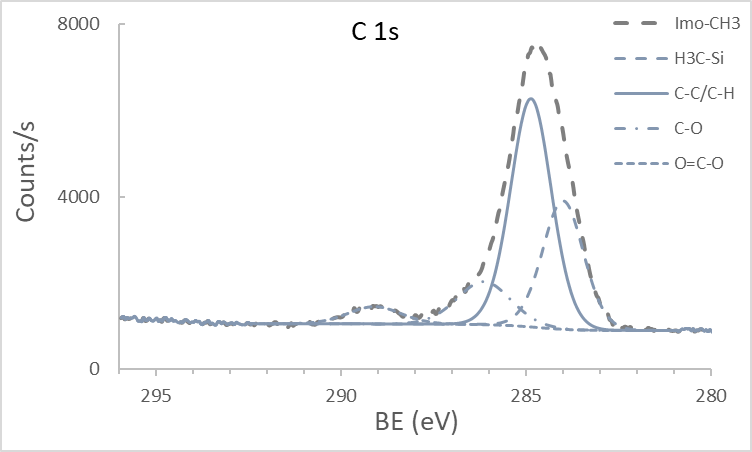 |
| --- | --- |

**Fig. S5**. Different contributions to C 1s core level spectra for Imo-OH (left) and Imo-CH_3_ (right) powders. C 1s signature comes mainly from contamination while for Imo-CH_3_ the Si-CH_3_ contribution is visible.

**Table S1.** Atomic quantification (%At) and identification (name and position) of each contribution to XPS spectra of Imo-OH and Imo-CH_3_ powders (extracted from the data presented in Fig. S2 to S5) using a calibration of Al 2p.

|  | name | position | %At |
| --- | --- | --- | --- |
| Imo-OH | O 1s | 531.79 | 52.02 |
|  | C 1S | 285 | 16.12 |
|  | C 1S | 286.48 | 2.39 |
|  | C 1S | 289.2 | 1.73 |
|  | Si 2P | 102.06 | 6.13 |
|  | Si 2p | 102.69 | 3.06 |
|  | Al 2p | 74.34 | 18.55 |
| Imo-CH_3_ | O 1s | 531.78 | 44.88 |
|  | C 1s | 284 | 8.51 |
|  | C 1s | 284.87 | 15.12 |
|  | C 1s | 286.14 | 3.76 |
|  | C 1s | 289.09 | 1.53 |
|  | Si 2p | 101.64 | 5.39 |
|  | Si 2p | 102.27 | 2.69 |
|  | Si 2p | 102.58 | 0.91 |
|  | Si 2p | 103.21 | 0.46 |
|  | Al 2p | 74.38 | 16.75 |


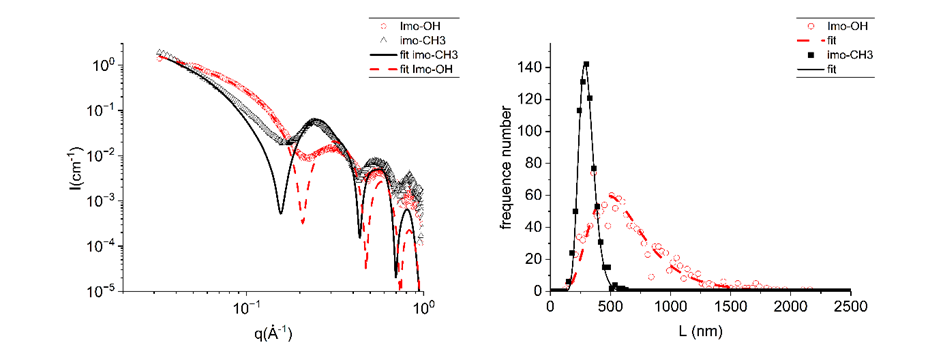


**Fig. S6.** Left) SAXS patterns of Imo-OH (red circle) and Imo-CH_3_ (black triangle) suspensions and fitting curves from monodisperse core-shell cylinder model (dashed and continuous lines for Imo-OH and Imo-CH_3_, respectively). The parameters are listed in Table S2. Right) length distribution of nanotubes extracted from AFM analysis (Imo-OH) or TEM analysis (Imo-CH_3_) with fit by lognormal distribution.

**Table S2.** Parameters used for the fitting of the SAXS patterns taking into account the formation of bundles.

|  | Inner diameter (nm) | Outer diameter (nm) | Internal electronic density (e^-^/Å^3^) | Number of associated tubes in bundles : 1, 2, 3 and 4 |
| --- | --- | --- | --- | --- |
| Imo-OH | 1.70 | 2.90 | 0.334 | (1, 0, 0, 0) |
| Imo-CH_3_ | 1.88 | 3.08 | 0.110 | (2, 0, 1, 1) |

**Table S3.** BAL fluid cell counts in mice following pulmonary exposure to 6, 18 and 54 μg of imogolites or 162 μg Printex90 (benchmark material). Data were obtained 1, 28 and 90 days post-exposure and shown as group mean ± SD. *: P ≤ 0.05, **: P ≤ 0.01, ***: P ≤ 0.001, ****: P ≤ 0.0001 compared to the vehicle control group. The green marking shows a statistically significant increase in cell numbers, whereas the orange marking is a statistically significant decrease in cell number. Data were analyzed by ordinary one-way ANOVA with Dunnett’s multiple comparison test.

| **Total number of cells (x 10^3^)** | | | | | |
| --- | --- | --- | --- | --- | --- |
| **Day 1** | 0 µg | 6 µg | 18 µg | 54 µg | 162 µg |
| Control | 47.2 ± 17.4 |  |  |  |  |
| Imo-OH |  | 86.4 ± 43.1* | 203 ± 56.0**** | 101 ± 35.7** |  |
| Imo-CH_3_ |  | 44.4 ± 20.3 | 59.0 ± 23.2 | 119 ± 42.1**** |  |
| Printex 90 |  |  |  |  | 153 ± 41.4**** |
|  | | | | | |
| **Day 28** | 0 µg | 6 µg | 18 µg | 54 µg | 162 µg |
| Control | 43.4 ± 10.6 |  |  |  |  |
| Imo-OH |  | 63.2 ± 20.4 | 97.0 ± 39.5*** | 130 ± 55.1**** |  |
| Imo-CH_3_ |  | 35.7 ± 16.4 | 42.0 ± 13.9 | 75.7 ± 39.5 |  |
| Printex 90 |  |  |  |  | 92.4 ± 35.0**** |
|  | | | | | |
| **Day 90** | 0 µg | 6 µg | 18 µg | 54 µg | 162 µg |
| Control | 43.1 ± 15.4 |  |  |  |  |
| Imo-OH |  | 42.1 ± 15.9 | 65.9 ± 8.99 | 90.6 ± 43.8**** |  |
| Imo-CH_3_ |  | 45.0 ± 11.1 | 47.0 ± 13.5 | 58.2 ± 5.07 |  |
| Printex 90 |  |  |  |  | 88.9 ± 28.6**** |

| **Macrophages (x 10^3^)** | | | | | |
| --- | --- | --- | --- | --- | --- |
| **Day 1** | 0 µg | 6 µg | 18 µg | 54 µg | 162 µg |
| Control | 40.2 ± 14.2 |  |  |  |  |
| Imo-OH |  | 28.6 ± 3.52 | 36.5 ± 20.2 | 14.1 ± 4.63*** |  |
| Imo-CH_3_ |  | 37.5 ± 15.0 | 29.1 ± 5.36 | 31.5 ± 8.88 |  |
| Printex 90 |  |  |  |  | 19.6 ± 15.9**** |
|  | | | | | |
| **Day 28** | 0 µg | 6 µg | 18 µg | 54 µg | 162 µg |
| Control | 41.2 ± 10.3 |  |  |  |  |
| Imo-OH |  | 47.6 ± 11.8 | 55.8 ± 19.1 | 80.1 ± 50.2*** |  |
| Imo-CH_3_ |  | 32.9 ± 17.4 | 42.7 ± 12.8 | 54.9 ± 11.0 |  |
| Printex 90 |  |  |  |  | 58.1 ± 19.9 |
|  | | | | | |
| **Day 90** | 0 µg | 6 µg | 18 µg | 54 µg | 162 µg |
| Control | 39.9 ± 12.2 |  |  |  |  |
| Imo-OH |  | 33.1 ± 8.95 | 46.7 ± 10.5 | 53.7 ± 17.6 |  |
| Imo-CH_3_ |  | 39.9 ± 8.35 | 47.2 ± 11.6 | 46.3 ± 8.78 |  |
| Printex 90 |  |  |  |  | 52.8 ± 19.1 |

| **Lymphocytes (x 10^3^)** | | | | | |
| --- | --- | --- | --- | --- | --- |
| **Day 1** | 0 µg | 6 µg | 18 µg | 54 µg | 162 µg |
| Control | 1.10 ± 1.04 |  |  |  |  |
| Imo-OH |  | 0.74 ± 0.92 | 7.46 ± 4.52**** | 1.41 ± 1.01 |  |
| Imo-CH_3_ |  | 0.26 ± 0.24 | 0.27 ± 0.17 | 1.50 ± 1.94 |  |
| Printex 90 |  |  |  |  | 6.87 ± 5.81**** |
|  | | | | | |
| **Day 28** | 0 µg | 6 µg | 18 µg | 54 µg | 162 µg |
| Control | 0.56 ± 0.66 |  |  |  |  |
| Imo-OH |  | 10.1 ± 15.2 | 27.6 ± 24.5** | 17.9 ± 21.3 |  |
| Imo-CH_3_ |  | 0.48 ± 0.67 | 1.49 ± 1.65 | 18.9 ± 31.8 |  |
| Printex 90 |  |  |  |  | 19.8 ± 17.9** |
|  | | | | | |
| **Day 90** | 0 µg | 6 µg | 18 µg | 54 µg | 162 µg |
| Control | 2.01 ± 21.6 |  |  |  |  |
| Imo-OH |  | 7.65 ± 7.46 | 14.9 ± 14.4 | 28.8 ± 20.6**** |  |
| Imo-CH_3_ |  | 4.32 ± 5.54 | 1.49 ± 0.84 | 6.52 ± 6.76 |  |
| Printex 90 |  |  |  |  | 28.8 ± 19.5**** |

| **Neutrophils (x 10^3^)** | | | | | |
| --- | --- | --- | --- | --- | --- |
| **Day 1** | 0 µg | 6 µg | 18 µg | 54 µg | 162 µg |
| Control | 5.05 ± 6.12 |  |  |  |  |
| Imo-OH |  | 48.5 ± 41.3** | 150 ± 47.0**** | 80.6 ± 30.9**** |  |
| Imo-CH_3_ |  | 4.72 ± 6.40 | 28.3 ± 19.8 | 80.9 ± 36.8**** |  |
| Printex 90 |  |  |  |  | 118 ± 39.0**** |
|  | | | | | |
| **Day 28** | 0 µg | 6 µg | 18 µg | 54 µg | 162 µg |
| Control | 0.68 ± 1.42 |  |  |  |  |
| Imo-OH |  | 2.44 ± 1.19 | 10.2 ± 5.25* | 27.8 ± 15.0**** |  |
| Imo-CH_3_ |  | 0.21 ± 0.20 | 1.05 ± 0.63 | 3.61 ± 2.31 |  |
| Printex 90 |  |  |  |  | 16.0 ± 9.70**** |
|  | | | | | |
| **Day 90** | 0 µg | 6 µg | 18 µg | 54 µg | 162 µg |
| Control | 0.83 ± 2.17 |  |  |  |  |
| Imo-OH |  | 0.73 ± 0.76 | 2.71 ± 2.13 | 7.87 ± 8.91*** |  |
| Imo-CH_3_ |  | 0.25 ± 0.34 | 0.37 ± 0.43 | 1.35 ± 1.20 |  |
| Printex 90 |  |  |  |  | 5.84 ± 4.85** |

| **Eosinophils (x 10^3^)** | | | | | |
| --- | --- | --- | --- | --- | --- |
| **Day 1** | 0 µg | 6 µg | 18 µg | 54 µg | 162 µg |
| Control | 0.36 ± 1.06 |  |  |  |  |
| Imo-OH |  | 6.28 ± 10.1 | 8.70 ± 5.28** | 4.43 ± 4.72 |  |
| Imo-CH_3_ |  | 0.36 ± 0.78 | 0.75 ± 1.14 | 3.69 ± 5.02 |  |
| Printex 90 |  |  |  |  | 8.15 ± 9.16**** |
|  | | | | | |
| **Day 28** | 0 µg | 6 µg | 18 µg | 54 µg | 162 µg |
| Control | 0.07 ± 0.19 |  |  |  |  |
| Imo-OH |  | 0.48 ± 1.18 | 0.07 ± 0.16 | 0.19 ± 0.47 |  |
| Imo-CH_3_ |  | 0.11 ± 0.24 | 0.17 ± 0.34 | 0.00 ± 0.00 |  |
| Printex 90 |  |  |  |  | 0.12 ± 0.27 |
|  | | | | | |
| **Day 90** | 0 µg | 6 µg | 18 µg | 54 µg | 162 µg |
| Control | 0.16 ± 0.42 |  |  |  |  |
| Imo-OH |  | 0.12 ± 0.30 | 0.00 ± 0.00 | 0.05 ± 0.14 |  |
| Imo-CH_3_ |  | 0.21 ± 0.35 | 0.00 ± 0.00 | 0.85 ± 1.76 |  |
| Printex 90 |  |  |  |  | 0.28 ± 0.43 |

**
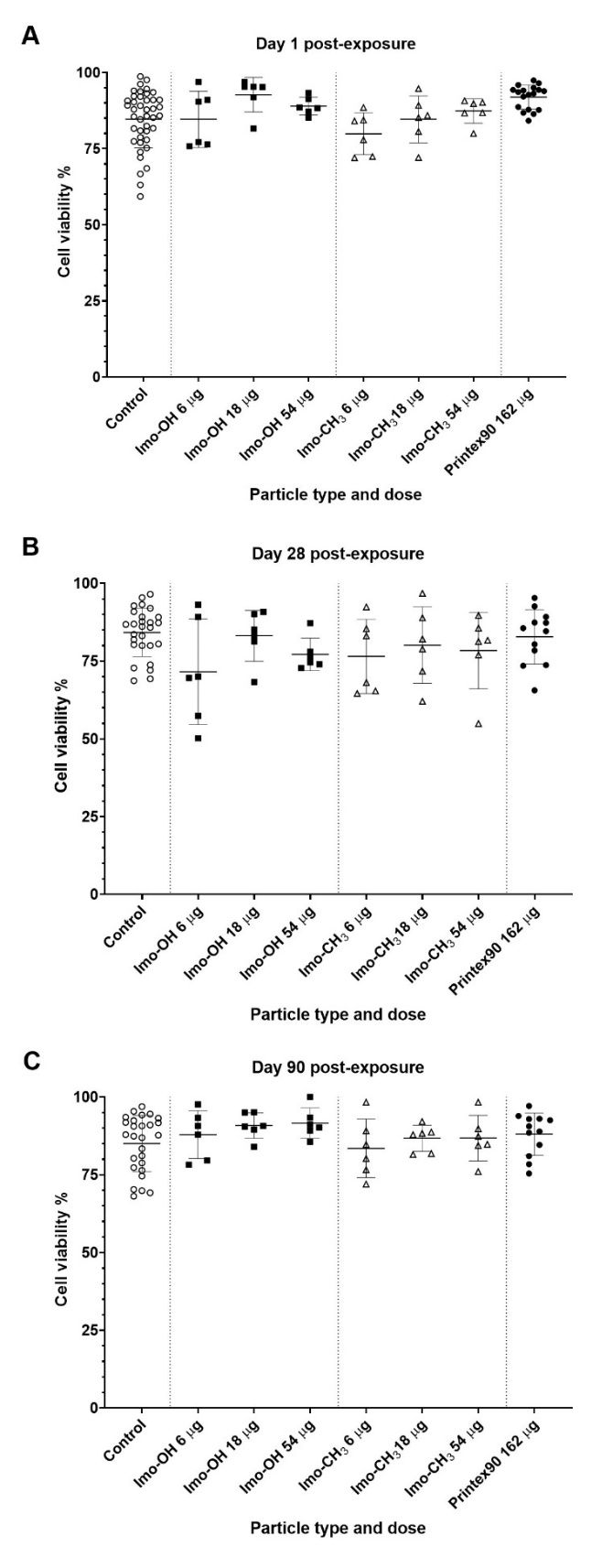
**

**Fig. S7.** BAL cell viability at 1 day (A), 28 days (B) and 90 days (C) post-exposure. Data are shown as individual data points (one per animal). Horizontal lines indicate the group mean ± SD. Group sizes (cumulative across overlapping series): day 1 − vehicle control (N = 40), Printex90 (N = 12), dose groups (n = 6 per group); days 28 and 90 − vehicle control (N = 27), Printex90 (N = 18), dose groups (n = 6 per group).

**
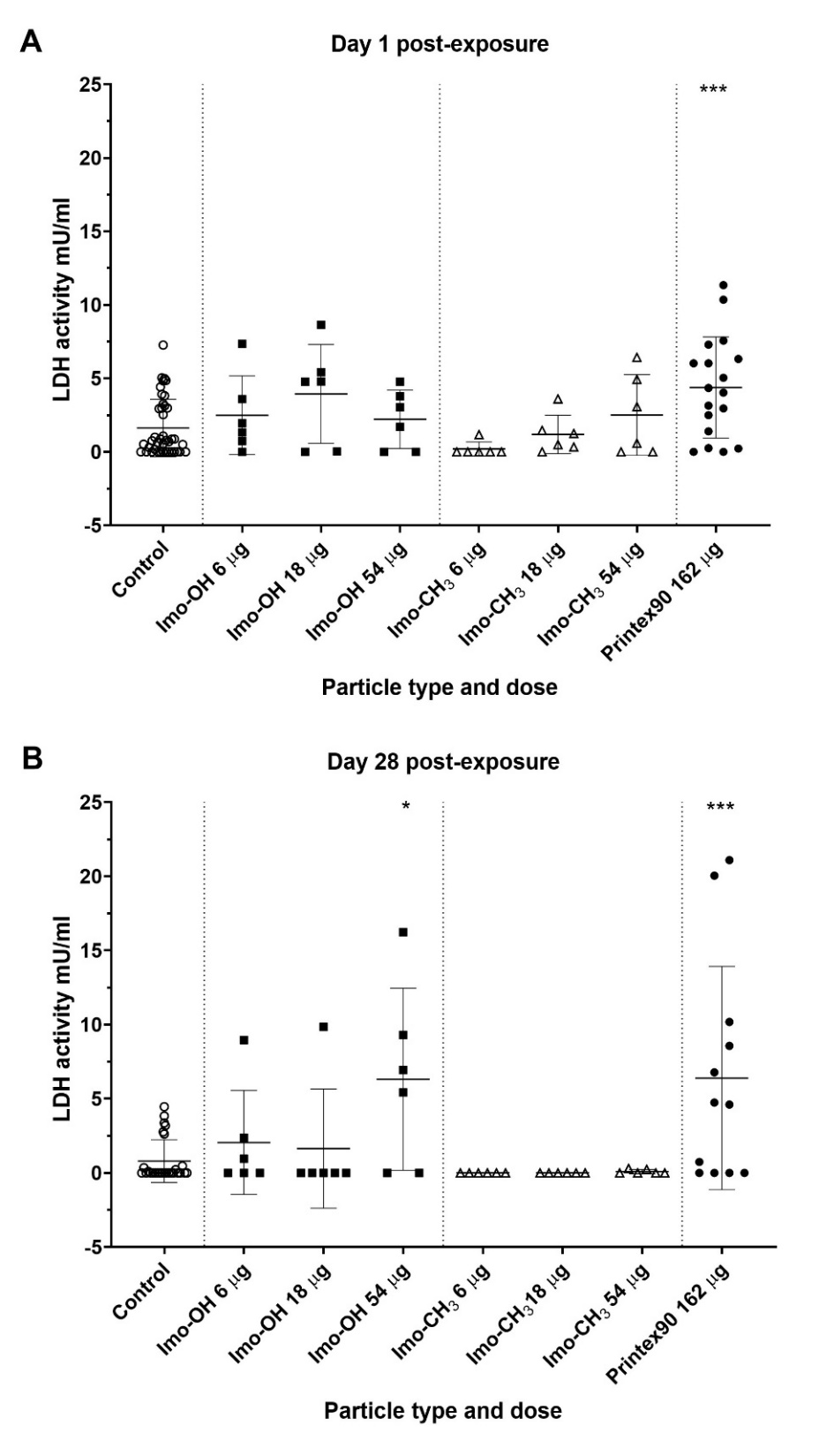
**

**Fig. S8.** LDH activity in BAL fluid at day 1 (A) and day 28 (B) post-exposure. Data are shown as individual data points (one per animal). Horizontal lines indicate the group mean ± SD. Group sizes (cumulative across overlapping series): day 1 − vehicle control (N = 40), Printex90 (N = 12), dose groups (n = 6 per group); day 28 − vehicle control (N = 27), Printex90 (N = 18), dose groups (n = 6 per group).


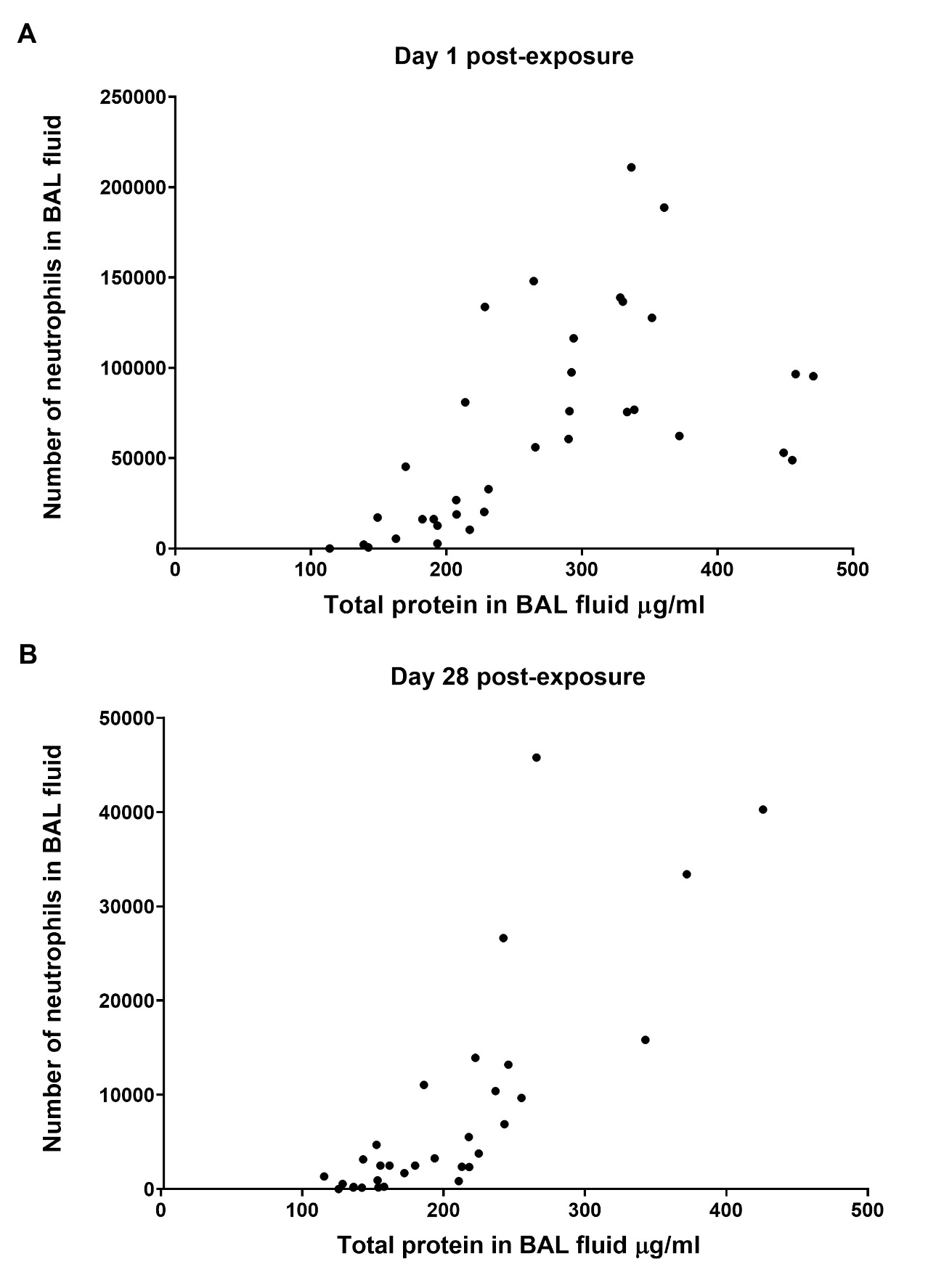


**Fig. S9.** Neutrophil influx and total protein in BAL fluid at 1 day (A) and 28 days (B) post-exposure. Data are shown as individual data points (one per animal). The six dose groups are included (n = 6 per group, N=36).


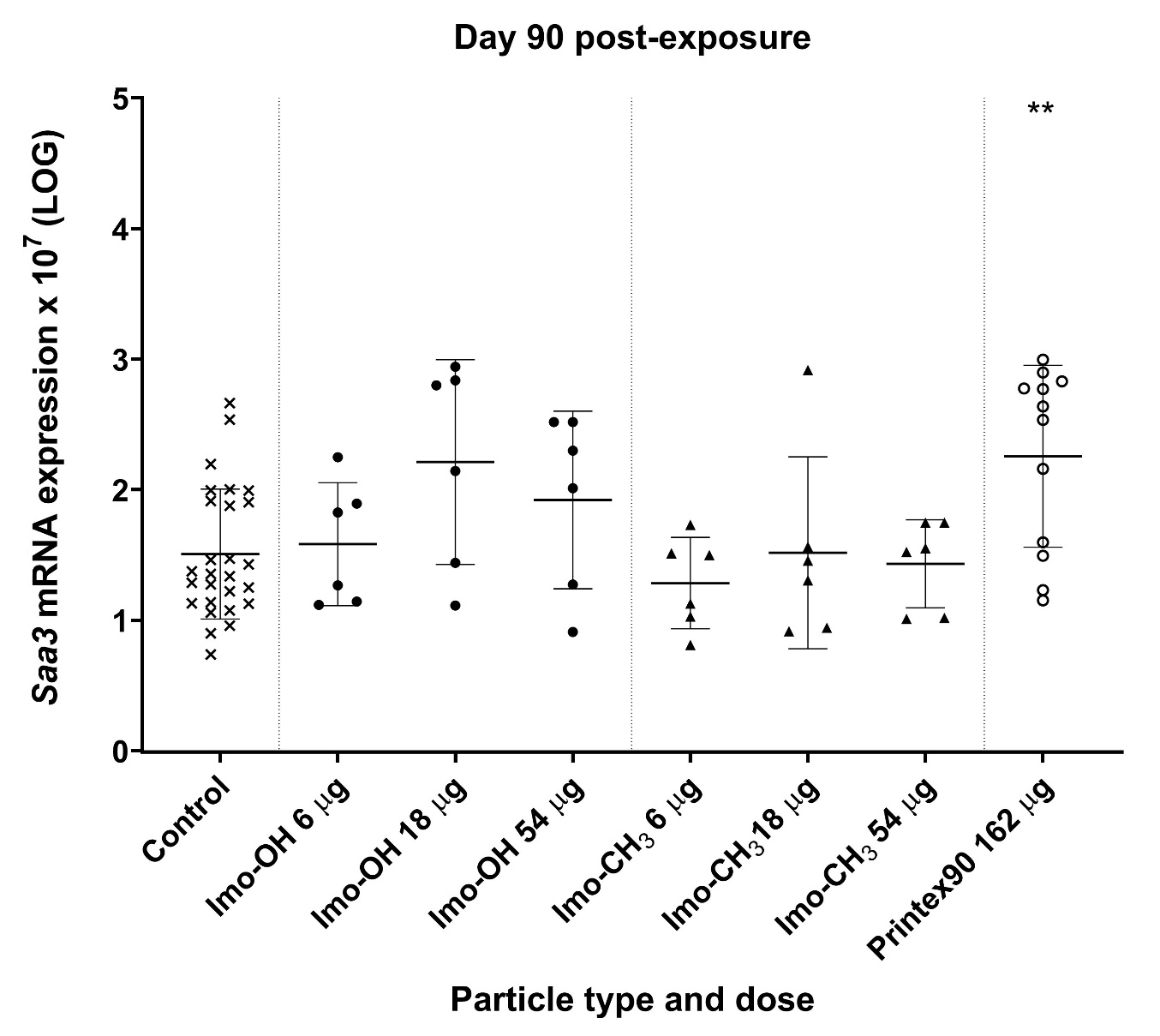


**Fig. S10**. Pulmonary *Saa3* mRNA expression levels at 90 days post-exposure. All values are log-transformed and presented as individual data points (one per animal). Horizontal lines indicate the group mean ± SD. Group sizes (cumulative across overlapping series): vehicle control (N = 27), Printex90 (N = 18), dose groups (n = 6 per group). ** denotes P ≤ 0.01 versus vehicle control (Dunnett’s multiple comparison method).


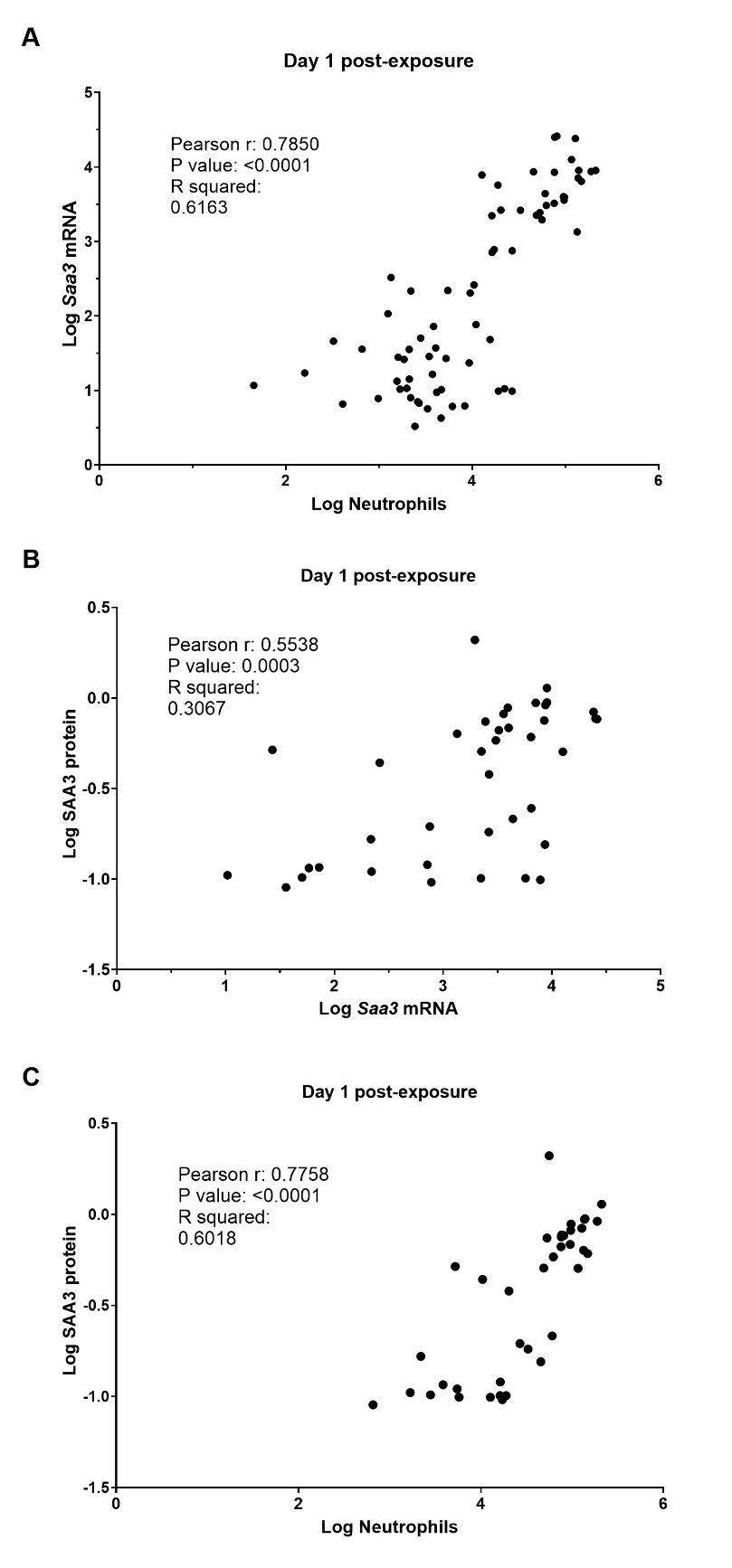


**Fig. S11.** Correlations between: (A) Neutrophil numbers and *Saa3* mRNA levels in lung tissue at 1 day post-exposure to imogolites; (B) *Saa3* mRNA levels in lung tissue and SAA3 plasma protein levels at 1 day post-exposure to imogolites; (C) Neutrophil numbers and SAA3 plasma protein levels at 1 day post-exposure to imogolites. Data are shown as individual data points (one per animal).


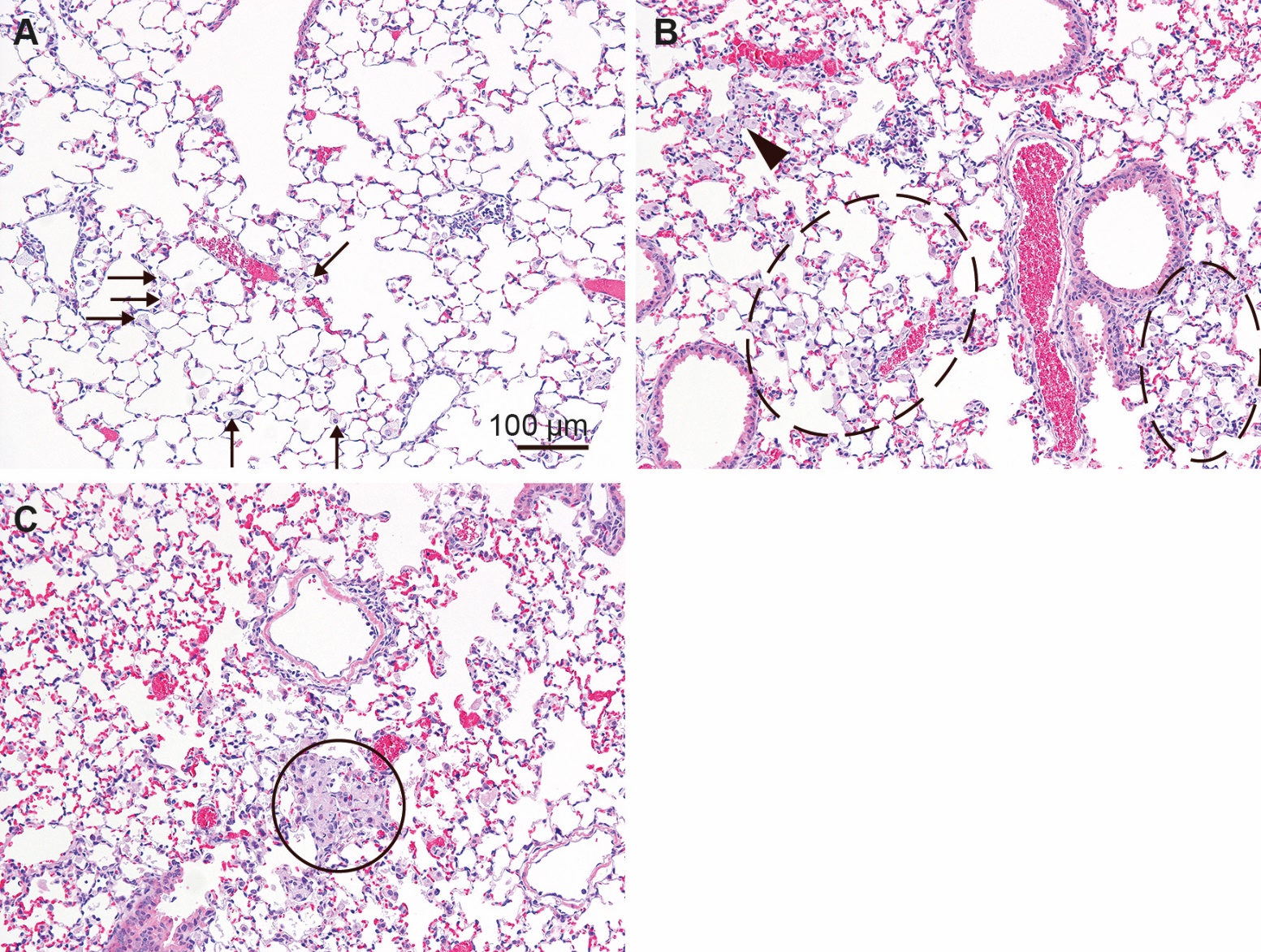


**Fig. S12.** Examples of (A) single laden macrophages (arrows), (B) an aggregate of laden macrophages (arrowhead) and areas of laden macrophages (ellipses), (C) an aggregate of “differentiated” macrophages (circle).

**Table S4.** Mononuclear cell infiltrations, laden macrophage aggregates and areas of laden macrophages in the lungs of mice 28 or 90 days after a single intratracheal instillation with a vehicle (controls) or with imogolites (54 µg/animal) Imo-OH or Imo-CH_3_. The number and severity of changes were reported separately for the left and the right lung (cranial, middle accessory, and caudal lobes)

| Day post exposure | | | |  | Day 28 | | | | |  | Day 90 | | | | | | | |
| --- | --- | --- | --- | --- | --- | --- | --- | --- | --- | --- | --- | --- | --- | --- | --- | --- | --- | --- |
|  | Type of change | Control  (n=6) | |  | Imo-OH  (n=5) | |  | Imo-CH_3_  (n=5) | |  | Control  (n=6) | |  | Imo-OH  (n=5) | |  | Imo-CH_3_  (n=5) | |
|  |  | Left | Right |  | Left | Right |  | Left | Right |  | Left | Right |  | Left | Right |  | Left | Right |
| Mononuclear cell infiltrations^A^ | | | | | | | | | | | | | | | | | | |
|  | Incidence^D^ | 0/6 | 1/6 |  | 4/5 | 5/5* |  | 3/5 | 5/5* |  | 1/6 | 3/6 |  | 4/5 | 5/5 |  | 2/5 | 4/5 |
|  | Total number per group | 0 | 1 |  | 19 | 103 |  | 9 | 27 |  | 2 | 13 |  | 58 | 75 |  | 7 | 28 |
|  | Mean number per group ± SD | 0.0±0.0 | 0.2±0.4 |  | 3.8±3.7 | 20.6±9.0 |  | 1.8±2.0 | 5.4±3.4 |  | 0.3±0.8 | 2.2±3.3 |  | 11.6±17.4 | 15±5.4 |  | 1.4±1.9 | 5.6±6.3 |
|  | Range | 0-0 | 0 -1 |  | 0-9 | 9-31 |  | 0-4 | 1-10 |  | 0-2 | 0-8 |  | 0-41 | 7-20 |  | 0-4 | 0-15 |
|  | Median | 0 | 0 |  | 3.0 | 23.0 |  | 1.0 | 5.0 |  | 0.0 | 0.5 |  | 2.0 | 17.0 |  | 0.0 | 2.0 |
|  | Multiplicity^E^ | 0 | 1 |  | 4.8 | 20.6 |  | 3.0 | 5.4 |  | 0 | 4.3 |  | 14.5 | 15.0 |  | 3.5 | 7.0 |
|  | Total severity^F^ score per group | 0 | 4 |  | 73 | 317 |  | 21 | 70 |  | 3 | 30 |  | 177 | 180 |  | 8 | 76 |
|  | Mean severity score per group ± SD | 0.0±0.0 | 0.7±1.6 |  | 14.6±13.0 | 63.4±34 |  | 4.2±5.0 | 14.0±8.9 |  | 0.5±0.1.2 | 5.0±7.5 |  | 35.4±61.4 | 36±16 |  | 1.6±2.3 | 15.2±18.9 |
|  | Severity range | 0-0 | 0-4 |  | 0-28 | 24-95 |  | 0-11 | 3-27 |  | 0-3 | 0-18 |  | 0-143 | 12-55 |  | 0-5 | 0-44 |
|  | Median severity per group | 0.0 | 0.0 |  | 15.0 | 73.0 |  | 2.0 | 15.0 |  | 0.0 | 1.0 |  | 3.0 | 35.0 |  | 0.0 | 4.0 |
| Laden macrophage aggregates ^B^ | | | | | | | | | | | | | | | | | | |
|  | Incidence | 0/6 | 0/6 |  | 3/5 | 5/5** |  | 0/5 | 3/5 |  | 0/6 | 0/6 |  | 3/5 | 3/5 |  | 0/5 | 0/5 |
|  | Total number per group | 0 | 0 |  | 10 | 40 |  | 0 | 13 |  | 0 | 0 |  | 8 | 12 |  | 0 | 0 |
|  | Mean number per group ± SD | 0.0±0.0 | 0.0±0.0 |  | 2.0 ± 2.0 | 8.0±7.4 |  | 0.0±0.0 | 2.6±3.4 |  | 0.0±0.0 | 0.0±0.0 |  | 1.6±2.1 | 2.4±2.5 |  | 0.0±0.0 | 0.0±0.0 |
|  | Range | 0-0 | 0-0 |  | 0-4 | 2-20 |  | 0 | 0-8 |  | 0-0 | 0-0 |  | 0-5 | 0-6 |  | 0 | 0 |
|  | Median | 0 | 0 |  | 2.0 | 5.0 |  | 0 | 1.0 |  | 0 | 0 |  | 1.0 | 3.0 |  | 0 | 0 |
|  | Multiplicity | 0 | 0 |  | 3.0 | 8.0 |  | 0 | 4.3 |  | 0 | 0 |  | 2.7 | 4.0 |  | 0 | 0 |
|  | Total severity score per group | 0 | 0 |  | 23 | 106 |  | 0 | 18 |  | 0 | 0 |  | 21 | 21 |  | 0 | 0 |
|  | Mean severity score per group ± SD | 0 | 0 |  | 4.6 ± 4.8 | 21.2±14.7 |  | 0 | 3.6±5.1 |  | 0.0±0.0 | 0.0±0.0 |  | 4.2±5.4 | 4.2±3.9 |  | 0 | 0 |
|  | Severity range between the animals in the group | 0-0 | 0-0 |  | 0-10 | 6-38 |  | 0-0 | 0-12 |  | 0-0 | 0-0 |  | 0-13 | 0-8 |  | 0-0 | 0-0 |
|  | Median severity per group | 0.0 | 0.0 |  | 4.0 | 18.0 |  | 0.0 | 1.0 |  | 0.0 | 0.0 |  | 3.0 | 6.0 |  | 0.0 | 0.0 |
| Areas of laden macrophages^C^ | | | | | | | | | | | | | | | | | | |
|  | Incidence | 0/6 | 0/6 |  | 4/5 | 4/5 |  | 4/5 | 5/5** |  | 0/6 | 1/6 |  | 4/5 | 5/5* |  | 3/5 | 3/5 |
|  | Total number per group | 0 | 0 |  | 10 | 42 |  | 8 | 15 |  | 0 | 1 |  | 11 | 30 |  | 5 | 10 |
|  | Mean number per group ± SD | 0.0±0.00 | 0.0±0.00 |  | 2.0±1.9 | 8.4±6.4 |  | 1.6±1.1 | 3.0±2.3 |  | 0.0±0.0 | 0.2±0.4 |  | 2.2±3.3 | 6.0±3.2 |  | 1.0±1.0 | 2.0±2.3 |
|  | Range | 0-0 | 0-0 |  | 0-5 | 0-14 |  | 0-3 | 0-6 |  | 0-0 | 0-1 |  | 0-8 | 2-9 |  | 0-2 | 0-5 |
|  | Median | 0 | 0 |  | 2.0 | 12.0 |  | 2.0 | 2.0 |  | 0 | 0 |  | 1 | 8.0 |  | 1.0 | 1.0 |
|  | Multiplicity | 0 | 0 |  | 2.5 | 10.5 |  | 2.0 | 3 |  | 0 | 1 |  | 2.8 | 6.0 |  | 1.7 | 3.3 |
|  | Total severity score per group | 0 |  |  | 29 | 105 |  | 15 | 37 |  | 0 | 1 |  | 37 | 53 |  | 8 | 28 |
|  | Mean severity score per group ± SD | 0 | 0 |  | 5.8±4.0 | 21.0±18.7 |  | 3.8±0.5 | 7.4±5.2 |  | 0.0±0.0 | 0.2±0.4 |  | 7.4±11.6 | 10.6±7.6 |  | 1.6±1.5 | 5.6±7.4 |
|  | Severity range | 0-0 | 0-0 |  | 0-10 | 0-47 |  | 0-4 | 0-16 |  | 0-0 | 0-1 |  | 0-28 | 2-18 |  | 0-3 | 0-16 |
|  | Median severity per group | 0 | 0 |  | 5.0 | 21.0 |  | 4.0 | 6.0 |  | 0 | 0 |  | 3.0 | 13.0 |  | 2.0 | 1.0 |

*: p>0.05, **: p<0.01 Fisher exact test on incidence data.

^A^: Mononuclear cell infiltrations: an area of lung tissue where the density of mononuclear cells is increased compared to the background of the surrounding, so that the mononuclear cells collection has a shape and size. Only the infiltrates of more than 20 cells were counted. The infiltrations consist usually of lymphocytes but other inflammatory cells like neutrophils or eosinophils can also be present in the infiltration. The infiltrations are observed near blood vessels or bronchiole or alveolar ducts, in the interstitium or subpleural.

^B^: Laden macrophage aggregates: more than three alveolar laden macrophages are seen at one site close to each other.

^C^: Area of laden macrophages: an area of tissue where the density of macrophages is higher than background. The term is used when the laden macrophages are situated not close to each other but oftest each in one alveolus.

^D^: Incidence is expressed as number of animals with the change of all animals in the group.

^E^: Multiplicity is expressed as a mean number of mononuclear cell infiltrations, of laden macrophage aggregates or of areas of laden macrophages for animals having a change in the group.

^F^: The severity of mononuclear cell infiltrations, of laden macrophage aggregates or of areas of laden macrophages was evaluated semi-quantitatively using a 5-grade system: grade 1: minimal/very few/very small; grade 2: mild/few/small; grade 3: moderate/moderate number/ moderate size; grade 4: marked/many/large; grade 5: massive/extensive number/extensive size. If the change was not present in the lungs of an animal a grade 0 was applied to indicate the absence. Each mononuclear cell infiltration, each laden macrophage aggregate or each area of laden macrophages was assigned a severity grade. Thereafter, a severity of the change was calculated as a total severity per group (sum of scores for all animals in the group), a mean severity score per group (sum of severity scores in the group / number of animals in the group) and a median. This was done separately for the left lung lobe referred as “Left” and for the right lung referred as “Right”, which consists of 4 lobes (cranial, middle, accessory and caudal). All five lobes were examined in all animals and the number and severity of mononuclear cell infiltrations, of laden macrophage aggregates or of areas of laden macrophages in each lobe was noted. The numbers for the right lung are obtained from all four lobes.

**Table S5.** Type and incidence of microscopic changes other than mononuclear cell infiltrations in the liver 28 or 90 days after a single intratracheal instillation with a vehicle (controls) or with imogolites (54 µg/animal) of Imo-OH or Imo-CH_3_.

| Day post exposure | Day 28 | | |  | Day 90 | | |
| --- | --- | --- | --- | --- | --- | --- | --- |
| Type of change | Control  n=6 | Imo-OH  n=5 | Imo-CH_3_  n=5 |  | Control  n=6 | Imo-OH  n=5 | Imo-CH_3_  n=5 |
| Necrosis (eosinophilic) of single hepatocyte adjacent to inflammatory cell infiltration | 0/6 | 0/5 | 0/5 |  | 1/6 | 1/5 | 1/5 |
| Apoptotic bodies in inflammatory cell infiltrations | 0/6 | 2/5 | 0/5 |  | 2/5 | 2/5 | 4/5 |
| Apoptosis of single hepatocytes (sporadically seen) | 0/6 | 0/5 | 0/5 |  | 0/6 | 0/5 | 1/5 |
| Steatosis microvesicular, minimal | 0/6 | 0/5 | 0/5 |  | 0/6 | 1/5 | 0/5 |
| Binucleate hepatocytes, apparent increase relative to controls, minimal | 0/6 | 0/5 | 3/5 |  | 0/6 | 0/5 | 0/5 |
| Polynucleate hepatocytes (sporadically seen) | 0/6 | 0/5 | 0/5 |  | 1/6 | 0/5 | 1/5 |
| Karyomegaly in single hepatocytes (sporadically seen) | 0/6 | 0/5 | 0/5 |  | 1/6 | 1/5 | 0/5 |
| Mitosis in single hepatocytes (sporadically seen) | 5/6 | 3/5 | 2/5 |  | 0/6 | 0/5 | 0/5 |
| Megakaryocyte observed | 0/6 | 2/5 | 0/5 |  | 1/6 | 0/5 | 0/5 |
| Kupffer cells, apparent increase relative to controls, minimal | 1/6 | 0/5 | 0/5 |  | 0/6 | 0/5 | 0/5 |
| Hyperplasia of connective tissue near bile ductules, minimal | 1/6 | 1/5 | 3/5 |  | 0/6 | 1/5 | 1/5 |
| Hyperplasia of oval cells, minimal | 0/6 | 0/5 | 1/5 |  | 0/6 | 0/5 | 0/5 |

^A^: Incidence of each change is expressed by the number of liver samples with a given change of total liver samples examined in the group.

**Table S6**. Focal mononuclear cell infiltrations in the liver of mice 28 or 90 days after a single intratracheal instillation with a vehicle (controls) or with imogolites (54 μg/animal) of Imo-OH or Imo-CH_3_.

| Focal mononuclear cell infiltration | | Day 28 | | |  | Day 90 | | |
| --- | --- | --- | --- | --- | --- | --- | --- | --- |
|  |  | Control  (n=6) | Imo-OH  (n=5) | Imo-CH_3_  (n=5) |  | Control  (n=6) | Imo-OH  (n=5) | Imo-CH_3_  (n=5) |
| Small | | | | | | | | |
|  | Incidence^A^ | 3/6 | 2/5 | 4/5 |  | 1/6 | 0/5 | 4/5 |
|  | Total number per group^B^ | 7 | 4 | 6 |  | 2 | 0 | 8 |
|  | Mean number per group^C^ ± SD | 1.2 ± 1.0 | 0.8 ± 1.3 | 1.2 ± 1.1 |  | 0.3 ± 0.8 | 0.0 ± 0.0 | 1.6 ± 1.5 |
|  | Range | 0 - 2 | 0 - 3 | 0 - 3 |  | 0 - 2 | 0 - 0 | 0-4 |
|  | Median | 1.5 | 0.0 | 0.0 |  | 0.0 | 0 | 1.0 |
|  | Multiplicity^D^ | 2.3 | 2.5 | 1.5 |  | 2 | 0 | 2.0 |
| Large | | | | | | | | |
|  | Incidence | 3/6 | 2/5 | 1/5 |  | 2/6 | 3/5 | 2/5 |
|  | Total number per group | 5 | 2 | 3 |  | 3 | 3 | 2 |
|  | Mean number per group ± SD | 0.8 ± 1.0 | 0.4 ± 0.5 | 0.6 ± 1.3 |  | 0.5 ± 0.8 | 0.6 ± 0.5 | 0.4 ± 0.5 |
|  | Range | 0-2 | 0 - 1 | 0 - 3 |  | 0-2 | 0 - 1 | 0-1 |
|  | Median | 0.5 | 0.0 | 0.0 |  | 0.0 | 1.0 | 0.0 |
|  | Multiplicity | 1.7 | 1.0 | 3.0 |  | 1.5 | 1.0 | 1.0 |
| Total (small and large) | | | | | | | | |
|  | Incidence | 4/6 | 4/5 | 4/5 |  | 3/6 | 3/5 | 5/5 |
|  | Total number per group | 12 | 6 | 9 |  | 5 | 3 | 10 |
|  | Mean number per group ± SD | 2.0 ± 1.7 | 1.2 ± 1.1 | 1.8 ± 1.6 |  | 0.8 ± 1.0 | 0.6 ± 0.5 | 2.0 ± 1.2 |
|  | Range | 0-4 | 0 - 3 | 0 - 4 |  | 0 - 2 | 0 - 1 | 1 - 4 |
|  | Median | 1.5 | 1.0 | 1.0 |  | 0.5 | 1.0 | 2.0 |
|  | Multiplicity | 3.0 | 1.5 | 2.3 |  | 1.7 | 1.0 | 2.0 |

^A^: Incidence of lesion is expressed by the number of livers with lesion of total number of examined.

^B^: Number of focal mononuclear cell infiltrations from all liver samples examined.

^C^: Mean number of focal mononuclear cell infiltrations for all liver samples examined.

^D^: Mean number of focal mononuclear cell infiltrations per group/numbers of liver samples with the given inflammatory cell infiltration in the group.
